# Dynamic Interplay between Structural Variations and 3D Genome Organization in Pancreatic Cancer

**DOI:** 10.1002/advs.202200818

**Published:** 2022-05-15

**Authors:** Yongxing Du, Zongting Gu, Zongze Li, Zan Yuan, Yue Zhao, Xiaohao Zheng, Xiaochen Bo, Hebing Chen, Chengfeng Wang

**Affiliations:** ^1^ Department of Pancreatic and Gastric Surgery National Cancer Center/Cancer Hospital Chinese Academy of Medical Sciences and Peking Union Medical College Beijing 100021 P. R. China; ^2^ Annoroad Gene Technology Co. Ltd Beijing 100176 P. R. China; ^3^ Department of Biotechnology Institute of Health Service and Transfusion Medicine Beijing 100850 P. R. China

**Keywords:** 3D genome, Hi‐C, pancreatic cancer, single‐molecule real‐time (SMRT) sequencing, structural variations

## Abstract

Structural variations (SVs) are the greatest source of variations in the genome and can lead to oncogenesis. However, the identification and interpretation of SVs in human cancer remain technologically challenging. Here, long‐read sequencing is first employed to depict the signatures of structural variations in carcinogenesis of human pancreatic ductal epithelium. Then widespread reprogramming of the 3D chromatin architecture is revealed by an in situ Hi‐C technique. Integrative analyses indicate that the distribution pattern of SVs among the 3D genome is highly cell‐type specific and the bulk remodeling effects of SVs in the chromatin organization partly depend on intercellular genomic heterogeneity. Meanwhile, contact domains tend to minimize these disrupting effects of SVs within local adjacent genomic regions to maintain overall stability. Notably, complex genomic rearrangements involving two key driver genes *CDKN2A* and *SMAD4* are identified, and their influence on the expression of oncogenes *MIR31HG*, *MYO5B*, etc., are further elucidated from both a linear view and 3D perspective. Overall, this work provides a genome‐wide resource and highlights the impact, complexity, and dynamicity of the interplay between structural variations and high‐order chromatin organization, which expands the current understanding of the pathogenesis of SVs in human cancer.

## Graphical abstract

This is the first report to study impacts of structural variations (SVs) on 3D genome organization in pancreatic cancer with multiomics strategies integrating long‐read sequencing and Hi‐C techniques. The findings provide a genome‐wide resource and new spatial perspective for comprehensively understanding the pathogenic mechanisms of SVs in pancreatic cancer development, which may contribute to identifying novel prognostic markers and therapeutic targets.

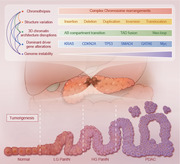

## 1 Introduction

Pancreatic cancer is one of the most lethal malignancies worldwide and ≈90% of cases involve pancreatic ductal adenocarcinoma (PDAC).^[^ ^1^ ^]^ Because these tumors are highly aggressive and metastatic, most patients are diagnosed at a late stage thus missing the opportunity to receive radical surgery, and chemotherapy or radiotherapy has limited effectiveness.^[^ ^2^ ^]^ With a gradually increasing incidence and little improvement in survival rate, PDAC is projected to be the second leading cause of cancer‐related death within a decade.^[^ ^3^ ^]^ Therefore, it seems that a significant improvement in pancreatic cancer mortality relies on the development of earlier detection and better treatment, which requires comprehensive knowledge of the molecular biology and pathogenesis of this disease.

Over the past few decades, the rapid development of next‐generation sequencing (NGS) technology (short‐read sequencing, SRS) has dramatically expanded our knowledge of genetic alterations, especially single‐nucleotide variations, in PDAC.^[^ ^4^ ^]^ While some recurrent gene mutations, such as *KRAS*, *CDKN2A*, *TP53*, and *SMAD4*, have been demonstrated to successively initiate and drive PDAC progression,^[^ ^5^ ^]^ the spectrum and pathogenesis of larger structural variations (SVs) in the context of pancreatic ductal epithelial cell carcinogenesis remain largely undefined due to technological limitations. SVs, including insertions, deletions, duplications, inversions, and translocations at least 50 bp in size, are the structural and quantitative chromosomal rearrangements that constitute the majority of genetic differences across human genomes.^[^ ^6^ ^]^ Accumulating evidence has demonstrated that SVs contribute to polymorphic variation, pathogenetic conditions, and many human diseases, such as cancers.^[^ ^7^, ^8^ ^]^ SVs can amplify oncogenes, delete tumor suppressor genes or affect noncoding genes involved in cancer susceptibility, thereby facilitating cancer genome evolution.^[^ ^9^, ^10^ ^]^ For a long time, SV detection has been performed through SRS approaches, which have been reported to lack sensitivity, exhibit a very high false‐positive rate and misinterpret complex or nested SVs.^[^ ^11^, ^12^ ^]^ Recently, long‐read methods, referred to as third‐generation sequencing (TGS) technologies, have been developed and shown to produce genome assemblies of unprecedented quality.^[^ ^13^, ^14^, ^15^ ^]^ The first true representative of TGS is single‐molecule real‐time (SMRT) sequencing, developed by Pacific Biosciences (PacBio).^[^ ^16^ ^]^ With average read lengths of 10 kbp or higher, reads can be more confidently aligned to the repetitive sequences that often mediate the formation of SVs. In addition, long reads are more likely to span SV breakpoints with high‐confidence alignments. These advantages highlight the need for third‐generation/long‐read sequencing of cancer genomes for the precise analysis of structural variant signatures to understand the molecular etiology underlying these diseases.^[^ ^17^, ^18^ ^]^

High‐throughput sequencing technologies can dramatically accelerate the discovery and characterization of SVs; however, the medical interpretation of SVs and the prediction of phenotypic consequences remain crucial challenges for geneticists and cancer biologists.^[^ ^19^ ^]^ Importantly, the discovery that SVs can be pathogenic without changing coding sequences indicated that SVs can not only directly alter gene expression through their effects on gene dosage,^[^ ^20^ ^]^ but also have regulatory effects by influencing the position and/or function of *cis*‐regulatory elements, such as promoters and enhancers, i.e., position effects.^[^ ^21^ ^]^ Owing to advances in 3D genome mapping technologies, such as high‐throughput chromosome conformation capture (Hi‐C) sequencing, it is now becoming increasingly evident that position effects are the result of alterations that are much more complex than simple changes in the linear genome and can be understood only by considering the 3D organization of the genome.^[^ ^10^ ^]^ Recent findings have indicated that dynamic changes in 3D genome architecture are associated with the development of multiple malignancies, including breast cancer,^[^ ^14^, ^15^, ^22^ ^]^ multiple myeloma,^[^ ^23^ ^]^ B cell lymphoma,^[^ ^24^ ^]^ and T cell acute lymphoblastic leukemia,^[^ ^25^, ^26^ ^]^ by coordinating the expression of some key driver genes. Notably, SVs can disrupt 3D genome organization and thereby exert indirect regulatory effects on gene expression.^[^ ^27^ ^]^ Meanwhile, the occurrence and formation of genomic rearrangements can be influenced by the 3D chromatin architecture,^[^ ^28^ ^]^ highlighting an action–reaction interplay between SVs and the 3D genome.

In light of these reports, understanding how SVs contribute to cancer pathogenesis by interplaying with chromatin organization remains largely unexplored. Herein, to broadly assess the global structural variation spectrum and 3D chromatin architecture in pancreatic cancer, we performed SMRT and in situ Hi‐C sequencing in two primary pancreatic ductal carcinoma cell lines (PANC1 and BxPC3) and an immortalized normal epithelial cell line (HPDE6C7). We further integrated the resulting datasets with transcriptomics by RNA‐seq to elucidate the influences of the dynamic interplay between SVs and 3D genome organization on gene expression. Moreover, some public datasets including enhancer activity, expressional level, and clinical prognosis were also acquired to validate the above regulatory correlation or biological significance. Our study provides fundamental new insights into the genetic and molecular basis of PDAC development and may contribute to the discovery of novel potential targets or biomarkers for precision therapy.

## 2 Results

### 2.1 The Signatures of Structural Variations in Human Pancreatic Cancer

**Figure 1:**
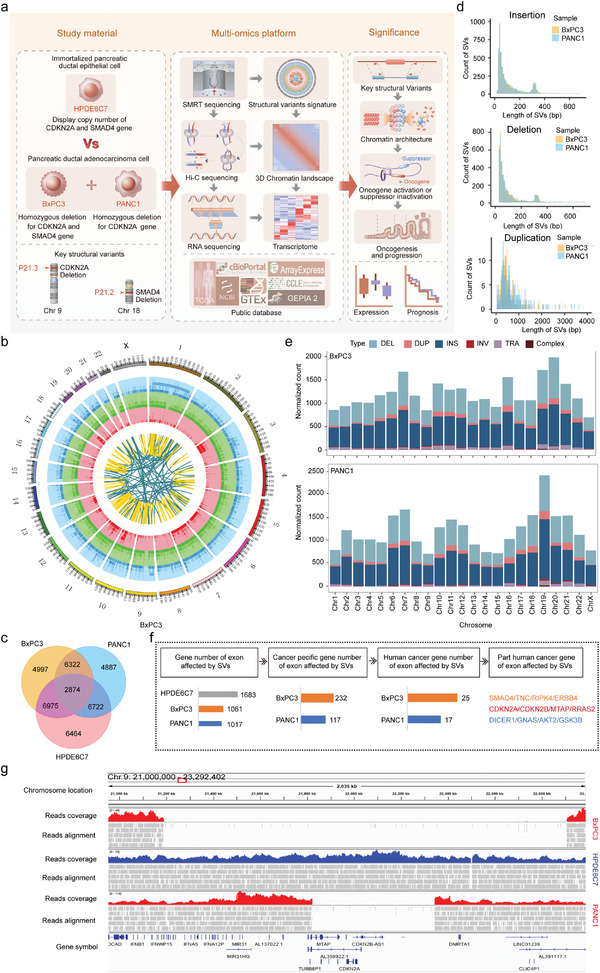
The overall landscape of SVs in PANC1, BxPC3, and HPDE6C7. a) Schematic diagram of the overall research design with the study material, multiomics platform, and significance presented. b) Circos plot showing the high‐confidence SVs detected by Sniffles in BxPC3 with 23 chromosomes inputted. The tracks from the outer to the inner circles are the chromosome coordinates, deletions, insertions, duplications, inversions, and translocations. c) Venn diagram showing the intersection of structural variations in two cancer cell lines (PANC1 and BxPC3) and one normal pancreatic ductal epithelial cell line (HPDE6C7) with counts indicated. d) Histograms showing the length distribution of specific SVs. e) Distribution of standardized total SV burden (deletion: light blue, duplication: pink, insertion: deep blue, inversion: red, translocation: light purple, complex: brown) across chromosomes. f) Pipeline of identification of specific cancer‐related genes directly affected by SVs in exonic regions in PANC1 and BxPC3. g) IGV image showing a homozygous deletion on chromosome 9 (covering *CDKN2A*, *CDKN2B*, and *MTAP*) of different lengths in BxPC3 and PANC1.

**Figure** **1**a shows the schematic diagram of the overall research design. We first comprehensively investigated the dynamic spectrums of SVs that occur during the malignant transformation of normal pancreatic ductal epithelial cells by SMRT sequencing. The structural variation data, processed by our SMRT‐bench platform, showed good alignment rates with a high percentage of usable long‐range read pairs (Figure S1 and Table S1, Supporting Information). By mapping to the reference genome GRCh37(hg19), a large number of SVs were detected, with total counts of 20 805, 21 168, and 23 035 SVs in PANC1, BxPC3, and HPDE6C7, respectively (Figure 1b and Figure S2a and Table S2, Supporting Information). The two most common types of SVs were insertions and deletions, which accounted for ≈50% and 41% of all SVs, respectively. Notably, the number of complex SVs in which more than two different types of simple SVs break‐ends overlapped was elevated two‐ to fourfold in cancer cell lines compared with normal epithelial cell line, which indicated that genome instability increased greatly during malignant transformation. Next, we explored the distribution of SVs in different regions of the genome and found that a majority of them were located in intergenic and intronic regions, as reported in previous studies^[^ ^10^, ^18^, ^29^ ^]^ (Figure S2b, Supporting Information).

To further identify the specific SVs that might lead to tumorigenesis, we compared all the SVs detected in cancer cells with those detected in normal epithelial cells and found that more than half of the SVs were specific to BxPC3 or PANC1 (Figure 1c and Table S3, Supporting Information). The number of common SVs shared by the three cell lines was 2874, which accounted for only 13% of all SVs detected, suggesting that SVs were polymorphic with high cell‐type specificity. Additionally, we studied the length distributions of different specific SV types. Insertions, deletions, and duplications showed similar characteristics, and most were within 1 kb in length (Figure 1d), while inversions and complex SVs were relatively different between the two cancer cell lines (Figure S2c, Supporting Information). In addition, we analyzed the standardized counts and proportions of different specific SV types within each chromosome. Although the normalized number of total SVs in each chromosome was slightly different, the proportion of each specific SV type was basically similar (Figure 1e).

Next, we further investigated the genes that were directly affected by SVs in their exon regions and obtained 1017, 1061, and 1683 genes in PANC1, BxPC3, and HPDE6C7, respectively (Figure 1f, and Table S4, Supporting Information). The specific genes affected by SVs in cancer cell lines were 177 and 232, of which 17 and 25 were reported as human cancer genes (HCGs) in the Catalogue of Somatic Mutations in Cancer (COSMIC, see URLs)^[^ ^30^, ^31^ ^]^ (Tables S5 and S6, Supporting Information). Notably, the specific HCGs shared by both cancer cell lines included *CDKN2A*, *CDKN2B*, and *MTAP* deletions on chromosome 9 and *RRAS2* insertions on chromosome 11. In addition, *DICER1* amplification on chromosome 14, *AKT2* deletion on chromosome 19, and *GNAS* insertion on chromosome 20 were independently detected in PANC1. *TNC* deletion on chromosome 9, *RRAS2* insertion on chromosome 11, and *SMAD4* deletion on chromosome 18 were specifically detected in BxPC3. *CDKN2A* homozygous deletion was demonstrated in PANC1 and BxPC3 but not HPDE6C7 in Cellosaurus, a knowledge resource for cell lines (see URLs, Table S7, Supporting Information). To confirm these findings in our data, the relevant genomes in three cell lines were visualized by IGV (Integrative Genomics Viewer, see URLs). There was an obvious large deletion in BxPC3 with a length of more than 1.7 Mb involving many genes in addition to *CDKN2A*, including *CDKN2B*, *MTAP*, and *DMRTA1* (Figure 1g). Similarly, a smaller deletion of ≈0.5 Mb that simultaneously covered the *CDKN2A*, *CDKN2B*, and *MTAP* genes was found in PANC1. Interestingly, we found another duplication in the region adjacent to this deletion in PANC1, which could also be verified by NGS data from the Yue Feng lab^[^ ^29^ ^]^ (Figure S2e, Supporting Information). In addition, a homozygous deletion of *SMAD4* was observed in BxPC3, which was consistent with data from Cellosaurus (Figure S2d, Supporting Information). These findings further supported the identity of our cell lines and the reliability of SV data generated using the SMRT‐bench platform in this study. The above SVs directly affected oncogenes or tumor suppressors in cancer cells and thus might play a significant role in the oncogenesis and maintenance of the malignant phenotype.

Together, these state‐of‐the‐art long‐read sequencing results establish signatures of SVs in human pancreatic cancer, which should provide a valuable resource for the comprehensive investigation of the pathogenesis of SVs in this deadly malignancy.

### 2.2 Widespread Remodeling of 3D Chromatin Architecture Correlates with Gene Expressional Changes in Human PDAC

To further reveal the impact of SVs on 3D chromatin architecture and gene expression, we applied in situ Hi‐C sequencing to comprehensively analyze the spatial conformation of chromosomes in the normal HPDE6C7 cell line and two cancer cell lines (BxPC3 and PANC1). Correlation analysis of the primary reads of three cell lines from different libraries indicated that Hi‐C data from different libraries were consistent and that the two cancer cell types were most similar to each other and could be distinguished from the normal HPDE6C7 cell line (Figure S3a and Table S8, Supporting Information). Next, we compared the 3D genome architecture of cancer cell lines with that of a normal epithelial cell line on multiple scales.

#### 2.2.1 Compartment Switching in PDAC

**Figure 2:**
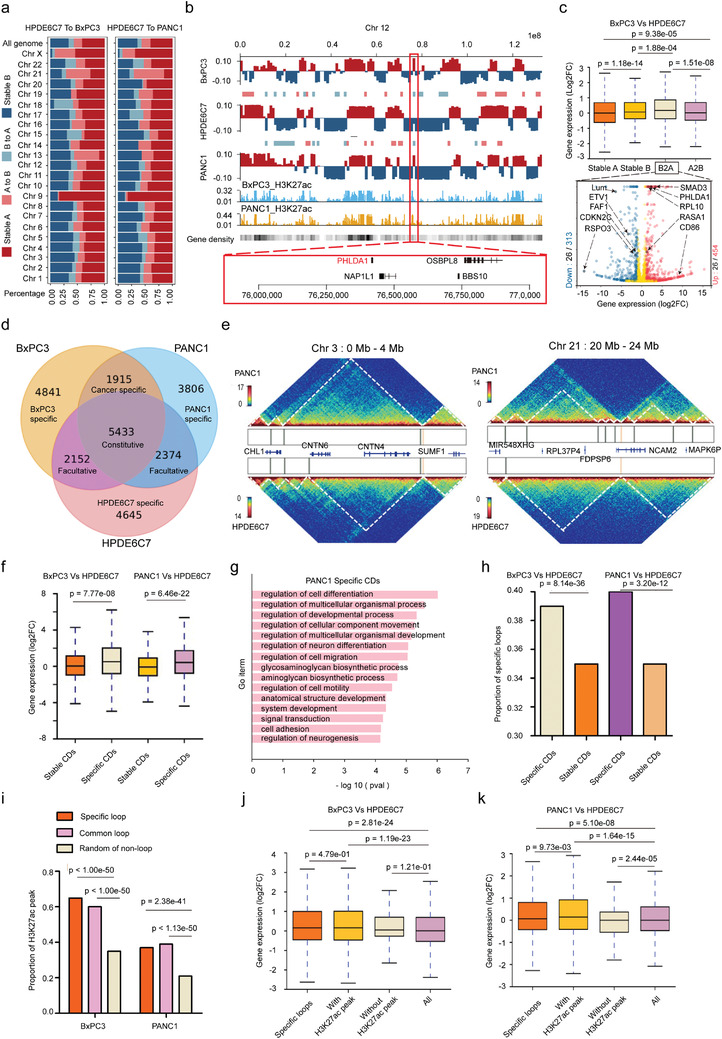
3D chromatin architecture remodeling correlates with gene expression changes in human PDAC. a) Compartment A/B switching of whole chromosomes in PANC1 and BxPC3 compared with HPDE6C7. Assignment of the A compartment (deep red) and B compartment (deep blue) was performed using eigenvalues > 0 and < 0, respectively. b) Examples of A/B compartment shifts on chromosome 12 in PANC1 and BxPC3 compared with HPDE6C7. Roadmap epigenome enhancer activity, marked by H3K27ac signal peaks, in PANC1 and BxPC3 is shown as blue and brown in histograms. Columns show the gene density in the genome. A red frame denotes a common B‐to‐A in both cancer cell lines covering the *PHLDA1* gene. c) Top: Box plots showing the comparison of gene expression levels in different compartments between BxPC3 and HPDE6C7. The box represents the interquartile range (IQR), the centerline denotes the median, and the whiskers extend to 1.5 times the IQR (or to the maximum/minimum if < 1.5 × IQR). Bottom: Volcano plots showing the number of differentially expressed genes (blue) and cancer‐related genes (black) among them in the B‐to‐A shift region. Genes indicated by the black arrow are examples of significantly upregulated (red on the right) or downregulated (blue on the left) cancer‐related genes (|Log2FC|>1 and adjusted *p* value < 0.05). Gene expression was compared as Log2FC (BxPC3/HPDE6C7) with the *p*‐value obtained by the Wilcoxon rank‐sum test. d) Venn diagram showing the intersection of CDBs obtained from the interaction matrices at 10 kb resolution in the three cell types with counts indicated. e) Examples of CD alterations in regions of interest (left chr 3: 0–4 Mb, right chr 21: 20–24 Mb) in PANC1 compared with HPDE6C7. The vertical bars in the box between heatmaps represent CDBs. Genes involved in the region are indicated. f) Box plots showing gene expression levels in different types of CDs in BXPC3 and PANC1 compared with HPDE6C7. The box represents the IQR, the centerline denotes the median, and the whiskers extend to 1.5 times the IQR (or to the maximum/minimum if < 1.5 × IQR). Gene expression was compared as Log2FC (cancer cell line/HPDE6C7) with the *p*‐value obtained by the Wilcoxon rank‐sum test. g) Biological process enrichment of differentially expressed genes located in specific CDs of PANC1. *p‐*Values were obtained by Fisher's exact test using EnrichR. h) Histograms representing proportions of specific loops in different types of CDs in BxPC3 and PANC1 compared with HPDE6C7. *p*‐Values were calculated by chi‐square test. i) Histograms represent the proportions of H3K27ac peaks in different types of loops in the genomes of BxPC3 and PANC1. *p*‐Values were calculated by chi‐square tests. j,k) Box plots represent the gene expression level in specific loops with or without H3K27ac peaks comparing BXPC3 and PANC1 with HPDE6C7. The box represents the IQR, the centerline denotes the median, and the whiskers extend to 1.5 times the IQR (or to the maximum/minimum if < 1.5 × IQR). Gene expression was compared as Log2FC (cancer cell line/HPDE6C7) with the *p*‐value obtained by the Wilcoxon rank‐sum test.

We first performed an analysis of compartment A/B transition in the genome and found that compared with HPDE6C7, the overall incidence of A/B switching was 24.8% in BxPC3 (A‐to‐B, 15.3%; B‐to‐A, 9.5%) and 24.1% in PANC1 (A‐to‐B, 16.2%; B‐to‐A, 7.9%), respectively. Although the percentage of A/B switching appeared different on each chromosome, the majority of the genome exhibited a stable A/B identity. Stable A was clearly the most common while A/B transition was the least part on chromosome 9 in both cancer cell lines (**Figure** **2**a and Table S8, Supporting Information).

Additionally, A/B switching was reported to be associated with changes in gene density and regulatory activity. Our data showed that stable A and B‐to‐A compartments were gene‐rich regions with active transcription, while stable B and A‐to‐B compartments had the opposite properties (taking chromosome 12 as an example in Figure 2b). Further analysis in combination with RNA‐seq data revealed that compartment A/B and compartment switching were significantly associated with gene expression changes, i.e., the gene expression in stable A and B‐to‐A compartments was significantly higher than that in stable B and A‐to‐B compartments (Figure 2c and Figure S4b, Supporting Information). Next, we analyzed the features of differentially expressed genes (|Log2FC| >1 and *q*‐value <0.05) and found that the number of differentially expressed genes in the A‐to‐B compartment was significantly higher than that in the B‐to‐A compartment in BxPC3/PANC1 cells (1291/1314 vs 767/696, *p* < 0.05) (Figure 2c and Figure S4b–d and Table S10, Supporting Information). We further aligned with the reported human cancer‐related genes and found that *PHLDA1*, a gene located in the common B‐to‐A compartment of chromosome 12, was significantly upregulated in both cancer cell lines (BxPC3:Log2FC 2.33, *q*‐value < 0.001; PANC1: Log2FC 1.94, *q*‐value < 0.001). Data from the GEPIA2 (gene expression profiling interactive analysis) server show that *PHLDA1* is significantly upregulated in several malignancies, including pancreatic cancer, lower‐grade glioma, and melanoma,^[^ ^32^ ^]^ and it is significantly associated with poor prognosis in pancreatic cancer.

Together, these data show that the spatial distribution of chromatin compartments A and B are changed in two cancer cell lines (BxPC3 and PANC1) compared with HPDE6C7, and these transitions were significantly associated with expression changes in cancer‐related genes.

#### 2.2.2 Contact Domain Alterations in PDAC

The topologically associating domain (TAD) is the functional unit of chromatin architecture. In previous studies, TADs were mainly identified by insulation score,^[^ ^23^, ^33^, ^34^ ^]^ but this method could identify only large topological units due to technical drawbacks leading to low resolution.^[^ ^35^ ^]^ To better characterize the contact domains (CDs) of normal and cancer cell lines, we employed high‐resolution Hi‐C, and contact domain boundaries (CDBs) were detected using the HiCDB method based on local relative insulation metrics and a multiscale aggregation approach on Hi‐C maps.^[^ ^36^ ^]^ We identified 14 581, 14 318, and 13 494 CDs in the HPDE6C7, BxPC3, and PANC1 cell lines with average sizes of 211, 227, and 214 kb, respectively (Figure S4e,f and Table S11, Supporting Information). Notably, most constitutive CDBs were shared by HPDE6C7, BxPC3, and PANC1 cells (21.59%), followed by cell‐specific CDBs (18.46%, 19.24%, and 15.12% for HPDE6C7, BxPC3, and PANC1, respectively). In contrast, few facultative CDBs were shared between any two cell types (8.55%, 7.61%, and 9.43% for HPDE6C7 vs BxPC3, BxPC3 vs PANC1, and PANC1 vs HPDE6C7, respectively) (Figure 2d and Table S12, Supporting Information). These results suggested that CDBs are conserved in the human genome.

For some cancer types (breast and prostate cancers, multiple myeloma), it has been reported that acquisition of new CD boundaries is usually accompanied by a corresponding increase in CD number and decrease in CD size.^[^ ^22^, ^23^, ^33^ ^]^ However, our study showed that the disappearance of CD boundaries was more typical in pancreatic cancer cells. Notably, changes in CD number and size could present diametrically opposite alterations in different chromosome regions. For example, on the Hi‐C map, fewer and longer CDs were observed in chromosome 3 of PANC1 than in the same region of HPDE6C7, while a region of chromosome 21 showed the opposite trend (Figure 2e); the same finding was also observed in BxPC3 (Figure S4g, Supporting Information). Similar results have been observed in gliomas and acute lymphoblastic leukemias,^[^ ^25^, ^37^ ^]^ suggesting that the trend of CD alterations in cancer is not absolute and that CDs may present quite diverse changes in different cancer types. Cancer heterogeneity and features specific to immortalized HPDE6C7 cells may be one of the contributing factors. To further explore the relationship between changes in different types of CDs and altered gene expression in the corresponding regions, we defined the newly emerging CDs in cancer cells as cancer‐specific CDs and the CDs shared with HPDE6C7 as stable CDs. We found that cancer‐specific CD regions were significantly more associated with upregulated gene expression compared with stable CDs (Figure 2f and Table S13, Supporting Information). Next, we analyzed expressional changes of genes in 1915 shared cancer‐specific CDBs. Interestingly, about 42% of genes level were found significantly different in both cancer cell lines PANC1(upregulated 337/1629 and downregulated 343/1629) and BxPC3 (upregulated 363/1629 and downregulated 313/1629) compared with HPDE6C7, including *TP53*, *SMAD3*, *PDGFA*, etc. (Table S14, Supporting Information). Further gene ontology (GO) analysis of differentially expressed genes in cancer‐specific CDs revealed that altered genes were involved in several key pathways, including cancer promotion, cell differentiation, cell adhesion, cell motility, and migration (Figure 2g and Figure S4h, Supporting Information).

#### 2.2.3 Cancer‐Specific Loops and Aberrant Enhancer Activations in PDAC

Chromatin loops were identified using Hi‐C Computational Unbiased Peak Search (HiCCUPS). If the observed/expected ratio of the two ends of the loops in the cancer cell line was twice as high as that in the normal control cell line, the loops were defined as cancer‐specific loops. To explore the relationship between differential gene expression and loops in chromatin, we profiled a total of 4046 and 1859 cancer‐specific loops in BxPC3 and PANC1, of which with H3K27ac peaks were 3743 and 1357, respectively (Table S15, Supporting Information). Further analysis of CDs revealed that cancer‐specific CDs were significantly associated with a high proportion of cancer‐specific loops (Figure 2h). Moreover, the proportion of active enhancers marked by H3K27ac was higher in cancer‐specific loops than in common loops and random nonloop regions in the genome (Figure 2i). And these cancer‐specific loops with H3K27ac activity were significantly associated with upregulated gene expression (Figure 2j,k). We further investigated these abnormally activated genes, which were associated with neo‐enhancer‐promoter loops. Totally, we obtained 967 and 238 significantly upregulated genes in BxPC3 and PANC1, respectively, including *TP63*, *LAMA3*, *CD58, MET*, etc. (Table S16, Supporting Information). These data suggested that the upregulation of gene expression in cancer‐specific CDs might be related to the activation of enhancers in cancer‐specific loops. Therefore, compared with normal HPDE6C7, spatial chromatin CDs are altered in cancer cell lines. Moreover, these changes are accompanied by upregulation of gene expression and may be related to the enhanced activity of regulatory elements in cancer‐specific loops.

In conclusion, the 3D chromatin architecture in PDAC cell lines has undergone widespread remodeling and consequent dysregulation of gene expression, which may promote tumorigenesis and progression of PDAC.

### 2.3 Distributions of Structural Variations among 3D Genome Architectures

Previous studies have indicated that the occurrence and formation of SVs are affected by 3D chromosome organization.^[^ ^28^ ^]^ Therefore, we next studied the distribution of the three most common types of SVs at the level of the 3D genome organization.

**Figure 3:**
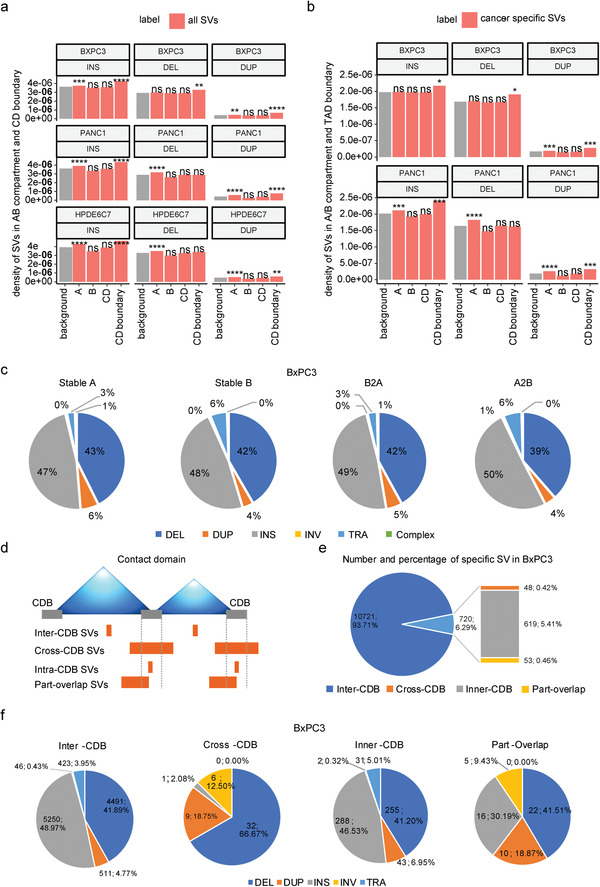
Distributions of structural variations among 3D genome architectures. a) Density of SVs (insertions, deletions, and duplications) in A/B compartments and CDBs. The density of SVs is the number of SVs divided by the length of each chromosome region. Gray bars represent background sequence and show the density of SVs on the chromosomes. Red bars show the density of SVs on the different chromosome regions, including A/B compartments, CDs, and CDBs. Enrichment tests were performed via R's prop. test, which was evaluated by comparing the proportion of SVs falling in the region of interest and the proportion of the length of the region of interest in the whole genome. ****p ≤ 0.0001, ***p ≤ 0.001, **p ≤ 0.01, *p ≤ 0.05. b) The density of cancer‐specific SVs (insertions, deletions, and duplications) in A/B compartments and TAD boundary. Cancer‐specific SVs refer to those occurring in BxPC3 (or PANC1) but not in HPDE6C7. Enrichment tests were performed via R's prop. test, which was evaluated by comparing the proportion of SVs falling in the region of interest and the proportion of the length of the region of interest in the whole genome. ****p ≤ 0.0001, ***p ≤ 0.001, **p ≤ 0.01, *p ≤ 0.05. c) The proportion of different types of cancer‐specific SVs (less than 2 Mb length) in different A/B compartment regions of BxPC3. d) Schematic diagram of SV categories according to the position relationship between the breakpoint of cancer‐specific SVs and the CD boundary. e) Number and percentage of four different specific SV types in BxPC3 according to (d). f) Number and percentage of different types of four categories of cancer‐specific SVs in BxPC3 according to (d).

We first explore the relationship between SVs and A/B compartments. Insertions, deletions, and duplications were all significantly enriched in the A compartment of BxPC3/PANC1/HPDE6C7 cells, except for deletions in BxPC3 (**Figure** **3**a). We next compared the A/B compartments of HPDE6C7 with those of BxPC3 or PANC1 and found that insertions, deletions, and duplications were significantly enriched among the stable A of BxPC3 and PANC1 (Figure S5a, Supporting Information). In addition, insertions, deletions, and duplications were significantly enriched in the A‐to‐B compartment in BxPC3, while deletions and duplications were significantly enriched in the B‐to‐A compartment in PANC1 (Figure S5a, Supporting Information), which indicated that the occurrence of SVs may be related to A/B compartment conversion. The differences in the distribution pattern among cell lines strongly suggest the cell‐type specificity of the correlation between SVs and A/B compartment transition.

Next, we selected cancer‐specific SVs from all SVs to study their distribution pattern in 3D chromatin architecture. Interestingly, we found that insertions, deletions, and duplications were significantly enriched in the A compartment in PANC1, but only duplications were enriched in the A compartment in BxPC3 (Figure 3b). In terms of the dynamic compartment transitions, insertions were significantly enriched in the A‐to‐B compartment in BxPC3, while they were only significantly enriched in the stable A compartment in PANC1; deletions were enriched in the stable A and A‐to‐B compartments in BxPC3, while they were enriched only in the stable A compartment in PANC1; the distribution of duplications was similar in BxPC3 and PANC1; and all duplications were enriched in the stable A compartment (Figure S5b, Supporting Information). The above findings indicated that the distribution patterns of cancer‐specific SVs in A/B compartments were quite different between the two cancer cell lines. Nevertheless, we further analyzed the compositions of different types of cancer‐specific SVs less than 2 Mb in length in A/B compartments in BxPC3 and PANC1 and found that insertions, deletions, and duplications accounted for approximately similar proportions in stable A, stable B, A‐to‐B, and B‐to‐A compartments in the two cancer cell lines (Figure 3c and Figure S5c and Tables S17 and S18, Supporting Information).

Next, we investigated the distributions of SVs in CDs and their boundaries. For all SVs, insertions and duplications were significantly enriched in the CD boundaries in BxPC3/PANC1/HPDE6C7, while deletions were enriched in the CD boundaries only in BxPC3 (Figure 3a and Figure S5d, Supporting Information). These findings indicated that most SVs tended to occur near the boundaries of CDs, consistent with previous studies.^[^ ^23^, ^38^ ^]^ We further classified CD boundaries by comparing HPDE6C7 with BxPC3 and PANC1 and found that insertions and duplications were significantly enriched only in the gained CD boundary in PANC1 but not in BxPC3 (Figure S5e, Supporting Information). Similarly, we observed the same phenomenon in cancer‐specific SVs (Figure S5f,g, Supporting Information), which indicated the significant cell‐type specificity of the degree of SV enrichment in CD boundaries.

Then, all cancer‐specific SVs were divided into four categories: inter‐CDB/cross‐CDB/inner‐CDB/part‐overlap according to the relative position between the breakpoint of SVs and CDB (Figure 3d). We found that more than 90% of cancer‐specific SVs were located inside the CDs (inter‐CDB‐SVs), which had little impact on their organization, while cross‐CDB/inner‐CDB/part‐overlap SVs, which had a greater probability of influence on CD folding, accounted for a relatively low proportion (Figure 3e and Figure S5h, Supporting Information). We further analyzed the compositions of different types of cancer‐specific SVs in the inter‐CDB/cross‐CDB/inner‐CDB/part‐overlap groups. Interestingly, the proportions of different types of cancer‐specific SVs in BxPC3 and PANC1 were roughly similar in the inter‐CDB/inner‐CDB/part‐overlap groups, while for the cross‐CDB group, the percentages of different types of SVs differed greatly (Figure 3f and Figure S5i and Table S19, Supporting Information).

In conclusion, there is a certain correlation between the occurrence of SVs and 3D chromosome organization in tumors at the A/B compartment or CD level. Furthermore, the distribution pattern of SVs among the 3D genome is highly cell‐type specific.

### 2.4 Interplay of Cancer‐Specific SVs and Chromatin Domains in PDAC Genomes

**Figure 4:**
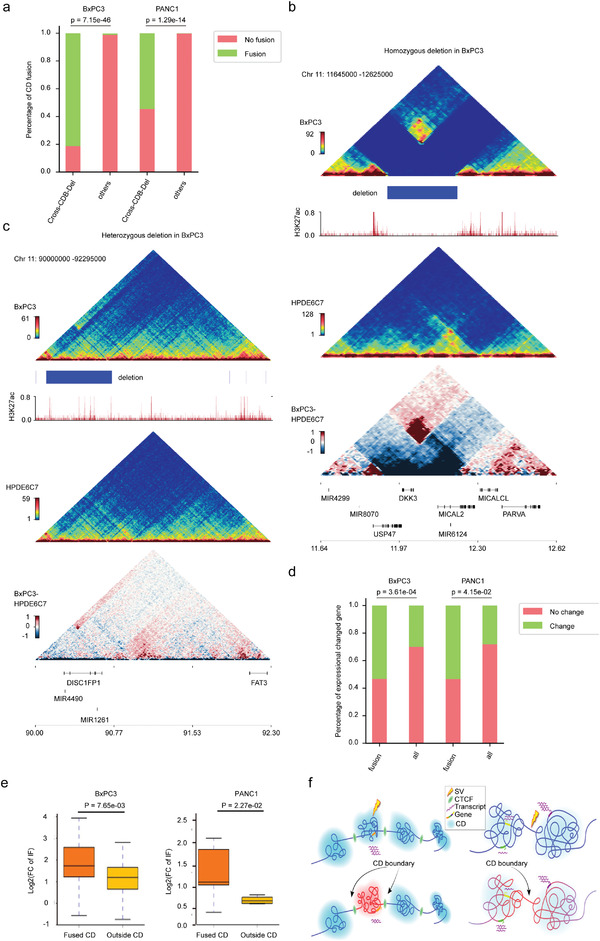
Cancer‐specific SVs affect gene regulation via reshaping CDs in PDAC genomes. a) Proportion of CD fusions in cross‐CDB deletion fields and other genome regions in BXPC3 and PANC1. *p*‐Values were calculated by Fisher's exact test. b,c) Examples of the impact of cross‐CDB deletion frequency on the chromatin folding domain in BXPC3. Triangle heatmaps represent chromatin contact frequency, with the top showing BxPC3, middle showing HPDE6C7, and bottom showing the subtractive results. Histogram representing roadmap epigenome enhancer activity, marked by H3K27ac, in BxPC3 (red). b) Homozygous cross‐CDB deletion is associated with CD fusion. c) No significant enhancement of adjacent CD interactions was observed at heterozygous cross‐CDB deletion. d) Proportion of differentially expressed genes in fused CDs and all genomes of BXPC3 and PANC1. *p*‐Value was calculated by Fisher's exact test. e) Box plots representing gene expression levels in fused CDs and outside CDs in BxPC3 and PANC1 cells. The box represents the IQR, with the centerline denoting the median; the whiskers extend to 1.5 times the IQR (or to the maximum/minimum if < 1.5 × IQR). Gene expression was compared as Log2FC (cancer cell line/HPDE6C7) with the *p*‐value obtained by the Wilcoxon rank‐sum test. f) Schematic diagram of the influence of SVs on gene expression in adjacent CDs. Left panel: The SV occurs within the CD, and the impact on gene regulation is generally restricted to this CD. Right panel: The SV occurs at a CD boundary or where CD structures are more loosely defined, and the effect on gene regulation spreads to adjacent CDs.

Previous studies have revealed that SVs can rewire chromatin organization to alter chromatin topologies and gene regulation in *cis*.^[^ ^10^, ^27^, ^39^ ^]^ To explore the impact of cross‐CDB SVs on CD disruption, we analyzed the correlation between cross‐CDB deletion and CD fusion and found that CD fusion was significantly more frequent in the cross‐CDB deletion regions than in other sites of the genome (**Figure** **4**a and Table S20, Supporting Information). These results indicate that cross‐CDB deletion is significantly associated with CD fusion, which is consistent with previous research results.^[^ ^10^, ^26^, ^40^ ^]^ However, not all cross‐CDB deletions could cause CD fusion, and further analysis found that only deletions with higher frequency in the same cell line were associated with enhanced interaction of adjacent CDs or CD fusion. Conversely, deletions with lower frequency had no significant effect on the interactions of adjacent CDs, and no CD fusion was identified in these cases (Figure 4b,c and Figure S6a,b, Supporting Information). Here, frequency refers to the percentage of SVs in the whole detected cell population. Obviously, higher frequency of deletions means less interaction in the relevant SV region on Hi‐C heatmaps. Similarly, cross‐CDB duplications did not always result in increased CD interactions; a significant increase in CD interactions or formation of neo‐CDs was observed at only a small number of cross‐CDB duplications that were homozygous or of higher copy number (Figure S7a,b, Supporting Information). These results indicate that the effects of SVs on the 3D genome architecture are quite complicated and may be influenced by multiple factors, such as the intercellular genomic heterogeneity, location, and length of the SVs.^[^ ^38^, ^41^ ^]^

We then analyzed the correlation between CD fusion and differential gene expression in two cancer cell lines. The results showed that the proportion of differentially expressed genes in fused CDs was significantly higher than that in other regions of the genome (Figure 4d and Tables S21 and S22, Supporting Information). More importantly, we further analyzed the interaction frequency of CDs adjacent to the cross‐CDB deletion and found that the interaction frequencies of fused CDs on either side of deletion were significantly higher than those of regions outside the fused CDs (Figure 4e), suggesting that the CD, as an essential functional unit of 3D chromatin, is able to confine the influences of SVs on 3D chromatin organization and gene expression to the adjacent CDs to maintain the structural and functional stability of the whole genome (Figure 4f).

Collectively, these data show that cancer‐specific SVs may regulate gene expression by remodeling CDs in PDAC. Moreover, the bulk remodeling effect observed in the 3D genome partly depends on intercellular genomic heterogeneity, which further expands our understanding of the pathogenesis of SVs in PDAC.

### 2.5 Impacts of *CDKN2A* Homozygous Deletion on 3D Genome Organization and Gene Expression

**Figure 5:**
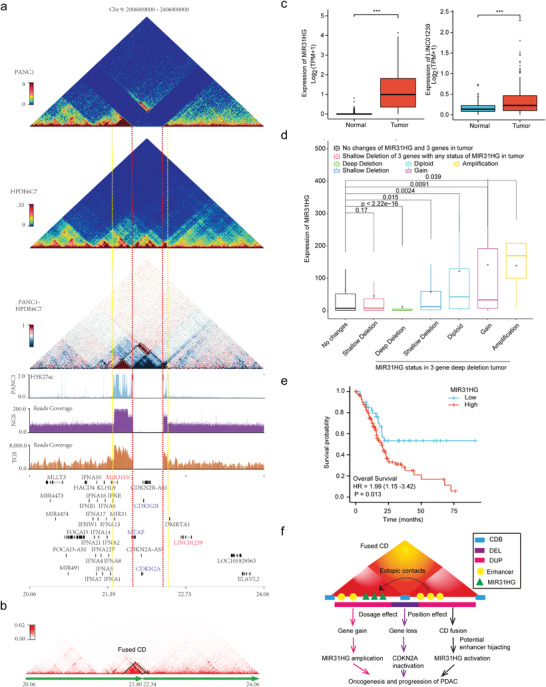
*CDKN2A* homozygous deletion is associated with MIR31HG upregulation partly through concomitant adjacent genome amplification and CD fusion. a) Diagram showing the impacts of *CDKN2A* homozygous deletion and concomitant amplification on 3D chromatin folding domains in PANC1. Triangle heatmaps represent chromatin contact frequency, with the top showing PANC1, middle showing HPDE6C7, and bottom showing the subtractive results. The histograms below represent the roadmap epigenome enhancer activity, marked by H3K27ac, in PANC1 (blue at top) and read coverage of next‐generation (purple at middle) and third‐generation (brown at bottom) sequencing for PANC1 in the same genomic region. The red dashed line denotes the breakpoints of the homozygous deletion, and the yellow dashed line marks the boundaries of the fused CD. The black dashed line in the bottom triangle heatmap indicates the enhanced internal and external interaction of the adjacent CD. b) Triangle heatmap showing the fused CDs with the black dashed line indicated. c) Expression of *MIR31HG* and *Linc01239* in pancreatic cancer and normal control tissues from TCGA and GTEx (*n* = 350). The box represents the IQR, the centerline denotes the median and the whiskers extend to 1.5 times the IQR (or to the maximum/minimum if < 1.5 × IQR). *p*‐Values were obtained by Wilcoxon rank‐sum test. ***p ≤ 0.001. d) *MIR31HG* expression levels under different mutation states of *MIR31HG* and three genes (*CDKN2A*, *CDKN2B*, and *MTAP*) in pancancer tissues from TCGA. The box represents the IQR, the centerline denotes the median and the whiskers extend to 1.5 times the IQR (or to the maximum/minimum if < 1.5 × IQR). *p*‐Values were obtained by Kruskal–Wallis test. e) Kaplan–Meier survival curves for overall survival according to *MIR31HG* expression in the TCGA pancreatic cancer dataset with a total of 178 cases (low group: 43, high group: 135). The *p*‐value was obtained by Cox regression in R (version 3.6.3). f) Schematic diagram showing that *CDKN2A* homozygous deletion could promote oncogenesis and the progression of PDAC by upregulating *MIR31HG* through concomitant amplification (dosage effect) and CD fusion (position effect). Dosage effects include oncogene MIR31HG amplification and suppressor *CDKN2A* inactivation. Position effects refer to potential enhancer hijacking through CD fusion.

*CDKN2A* inactivation occurs in ≈90% of PDACs through various mechanisms, among which homozygous deletion is one of the most common pathways.^[^ ^2^, ^42^, ^43^ ^]^ Our above findings confirmed the homozygous deletion of *CDKN2A* in both BxPC3 and PANC1 (Figure 1g). Next, we explored the effect of this homozygous deletion on 3D genome organization and gene expression in chromosome 9. Given the differences in deletion length between the two cancer cell lines, we first carried out the analysis in PANC1 and found that the interaction between adjacent CDs on both sides of the deletion was significantly enhanced to form a fused CD. Moreover, the internal interaction was also significantly intensified between adjacent CD regions, which was consistent with the finding of duplications on both sides of this deletion in the genomes by TGS and NGS (**Figure** **5**a,b). We further analyzed the gene expression changes in this region and found that the expression of *CDKN2A*, *CDKN2B*, and *MTAP* (genes in the deletion region) had almost disappeared, whereas the expression of *MIR31HG* and *LINC01239* (genes on either flanking region of the deletion) had been significantly upregulated (Figure S8a and Table S23, Supporting Information). However, there were no significant changes in the expression of IFNA family members, which may be attributed to the lack of transcriptional activity of alpha interferon in both cell lines without further stimulation by viral infection. Interestingly, we also found the *MTAP‐DMRTA1* gene fusion, which was consistent with the previous NGS results of PANC1 in the CBioPortal database. Both *MIR31HG* and *LINC01239* are long noncoding RNAs, and data from TCGA and GTEx revealed that their expression was significantly higher in PDAC tissues than in adjacent normal tissues (Figure 5c). Previous studies have shown that *MIR31HG* presents a carcinogenic phenotype in various solid tumors, such as PDAC, squamous cell carcinoma of the head and neck, and esophageal cancer,^[^ ^44^, ^45^, ^46^ ^]^ while the effects of *LINC01239* have rarely been reported. As *MIR31HG* is located in the fused CDs, it can be speculated that the upregulation of *MIR31HG* in PANC1 might be related to the *CDKN2A*, *CDKN2B*, and partial *MTAP* deletion in combination with the amplification of adjacent genome regions on both sides. Next, we analyzed the correlation between *MIR31HG* expression and the deletion of these three genes (*CDKN2A*, *CDKN2B*, and *MTAP*) in 807 cancer cell lines from the CCLE database. Although *MIR31HG* expression was not significantly upregulated in cells with the three‐gene deletion and *MIR31HG* diploidy or amplification compared with cells in which *MIR31HG* and all three genes were diploid, an upward trend of *MIR31HG* expression could still be observed in the amplification group (Figure S8b,c and Table S24, Supporting Information); the lack of statistical significance may be related to the small sample sizes of the two groups. Therefore, we further analyzed 8359 pan‐cancer samples from TCGA database and found that *MIR31HG* expression under different *MIR31HG* mutation statuses in cancer tissues with deep deletion of the three genes (*CDKN2A*, *CDKN2B*, and *MTAP*) was significantly different from that in cancer tissues in which all *MIR31HG* and three genes were diploid. Notably, *MIR31HG* expression was significantly increased in *MIR31HG* diploid and amplified cancer tissues, indicating that the upregulation of *MIR31HG* expression was significantly correlated with *MIR31HG* copy number amplification and *CDKN2A‐CDKN2B‐MTAP* deletion in a pan‐cancer sample (Figure 5d and Figure S8d and Table S25, Supporting Information). In addition, we also analyzed the impact of *MIR31HG* expression on the prognosis of patients with PDAC and found that the survival of patients with high *MIR31HG* expression was significantly shortened (Figure 5e), consistent with the carcinogenic role of *MIR31HG* in PDAC.

Similarly, we studied the homozygous deletion related to *CDKN2A* in BxPC3. As the length of deletion was larger than that in PANC1, the expression of the *CDKNA2A*, *CDKN2B*, *MTAP*, and *MIR31HG* genes, which were within the range of the deletion, was lost. At the same time, the interaction between adjacent CDs on both sides of this deletion was significantly enhanced, forming a CD fusion. However, due to the lack of expressed genes in the fused CD region, no changes in expression were observed (Figure S8a,e, Supporting Information).

These findings suggested that *CDKN2A* homozygous deletion was associated with upregulation of *MIR31HG* expression in PDAC, which may be related to concomitant amplification and CD fusion in the adjacent genomic regions of *CDKN2A* homozygous deletion (Figure 5f). In conclusion, our research revealed the effects of *CDKN2A* homozygous deletion on 3D genome organization and gene expression, providing new insight for understanding *CDKN2A* inactivation to drive the occurrence and development of PDAC.

### 2.6 Identification of Complex Genomic Rearrangements Involving *SMAD4* Deletion and Their Influence on 3D Genome and Gene Expression

**Figure 6:**
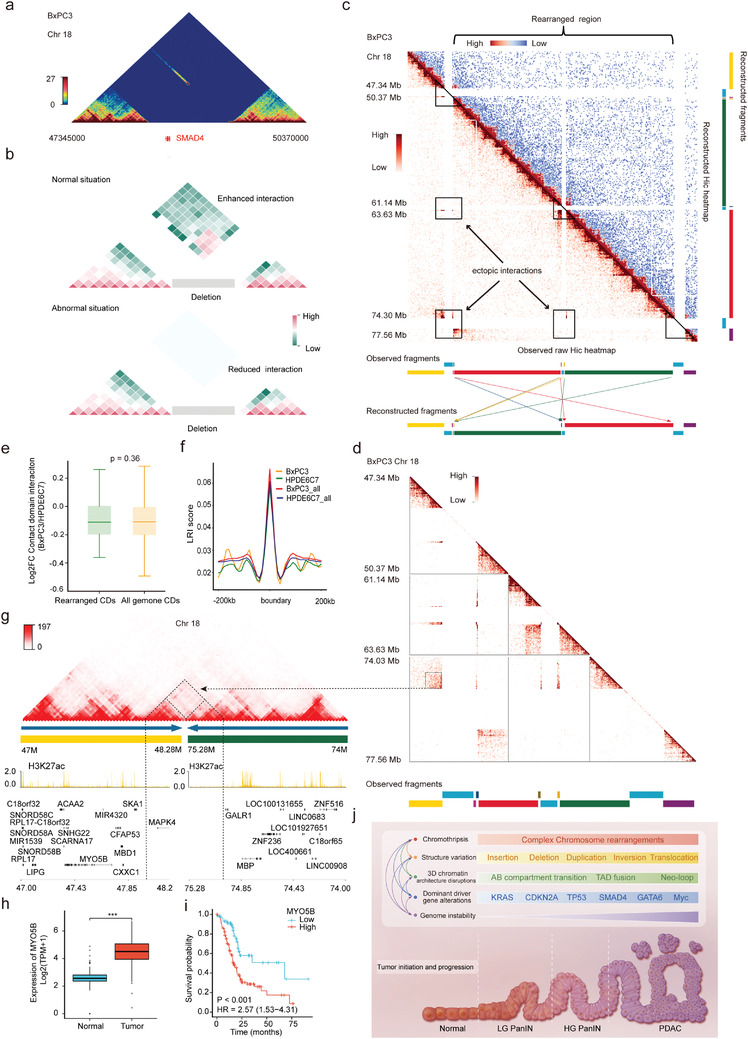
Identification of complex genomic rearrangements associated with *SMAD4* deletion in PDAC. a) Triangle heatmap showing the interaction on both sides of the cross‐CDB deletion involving *SMAD4* in BxPC3. No interaction was observed on adjacent CDs of this homozygous deletion except for a single bin at 40 kb resolution. b) Schematic diagrams showing the impacts of homozygous cross‐CDB deletion on the interaction of adjacent CDs. Top: The interaction on both sides of the cross‐CDB deletion is enhanced in most normal cases. Bottom: The interaction on both sides of the cross‐CDB deletion is reduced in some abnormal situations. c) Observed interaction heatmaps (lower‐left triangle, interaction reads mapping to hg19 reference genome) and reconstructed heatmap (upper‐right triangle) of chromosome 18 in the BxPC3 cell line. *SMAD4* is involved in the first region marked with a triangle on the left. The aberrant chromosomal long‐range interactions (indicated by black rectangles) all but disappeared after the rearrangement. Segments with different colors represent genome fragments. Arrows in different colors show simplified rearranged models of relevant segments with the same color. Briefly, red and green segments were translocated and inverted, and small segments beside them were also translocated after reconstruction. d) Amplification of regional heatmaps indicated by triangles and rectangles in (c). The dashed box shows the aberrant enhanced interaction on the junction region of rearranged genome fragments corresponding to new contact domain (neo‐CD) in (g). e) Box plots representing the comparison of interaction frequency between CDs in the rearranged region and in the whole genome. Interaction frequency was reported as Log2FC (BxPC3/HPDE6C7). The box represents the IQR, the centerline denotes the median and the whiskers extend to 1.5 times the IQR (or to the maximum/minimum if < 1.5 × IQR). *p*‐Values were obtained by Wilcoxon rank‐sum test. f) Comparison of the local relative insulation (LRI) score of the CDB in the rearranged region (yellow: BxPC3, green: HPDE6C7) and all genomes (red: BxPC3, blue: HPDE6C7). g) Example of neo‐CD formation in the junction region of rearranged yellow and green genome fragments in chromosome 18 of BxPC3 by NeoLoopFinder. The dashed triangle denotes the neo‐CD corresponding to ectopic interactions in (d) with the dashed black arrow indicated. Histograms below represent roadmap epigenome enhancer activity, marked by H3K27ac, in BxPC3 (yellow). h) Expression of *MYO5B* in pancreatic cancer and normal control tissues from TCGA and GTEx (*n* = 350). The box represents the IQR, the centerline denotes the median and the whiskers extend to 1.5 times the IQR (or to the maximum/minimum if < 1.5 × IQR). *p*‐Values were obtained by Wilcoxon rank‐sum test. ***p ≤ 0.001. i) Kaplan–Meier survival curves for overall survival according to *MYO5B* expression in the TCGA pancreatic cancer dataset with a total of 178 cases (low group: 62, high group: 116). *p*‐Values were obtained by Cox regression in R (version 3.6.3). j) Schematic diagram showing that multiple molecular biological events are involved in the carcinogenesis and progression of pancreatic cancer. These events are not independent of each other but rather engage in crosstalk and generate a complex regulatory network.

*SMAD4*, one key driver gene of PDAC, is known to be lost in ≈55% of pancreatic cancers, with homozygous deletion accounting for ≈30% of these cases.^[^ ^47^ ^]^ However, little is known about the effect of homozygous *SMAD4* deletion on 3D genome organization. The SMAD4 homozygous deletion in BxPC3 was validated by both the TGS technique and the Hi‐C method (**Figure** **6**a and Figure S2d, Supporting Information). According to our abovementioned findings, cross‐CDB deletion enhanced adjacent CD interactions on both sides of the breakpoints (Model Figure 6b‐top). Surprisingly, the interaction between the two sides of the cross‐CDB deletion involving the *SMAD4* gene was not enhanced but disappeared, and only a single bin on both sides of the deletion was found to have enhanced interaction at 40 kb resolution (Figure 6a, Model Figure 6b‐bottom). To further explore the ground truth underlying these abnormal changes, we first checked the interaction heatmap of chromosome 18 of BxPC3 and found several enhanced distal ectopic interactions. These unusual long‐range regions were mainly related to three large deletion sites, including the region of *SMAD4* deletion (Figure 6c). Next, we identified the three large deletion sites from 40 kb Hi‐C matrices and divided chromosome 18 into scaffolds (Figure 6c,d). Then, we rearranged the scaffolds to map them to the new chromosome 18 according to de novo genome assemblies based on chromatin interactions.^[^ ^48^ ^]^ The aberrant long‐range chromosomal interactions all but disappeared after the rearrangement. These results indicate that homozygous loss of *SMAD4* and multiple deletions on chromosome 18 in BxPC3 lead to huge, complex chromosomal rearrangements, including inversions and translocations. This is consistent with previous reports of chromothripsis in the same regions in ≈11% of pancreatic cancer cases^[^ ^4^ ^]^ and maybe the main cause of the paradoxical changes in local chromatin interactions. Interestingly, the chromothripsis and reorganization of chromosome 18 in BxPC3 seem to be homozygous. This may indicate that there exists some special preference mechanism for chromosome reconnection.

Then, we found that the interaction frequencies of CDs in the chromosomal 18 rearrangement region did not change significantly and that the CDB remained basically unchanged (Figure 6e,f), which is consistent with the conserved nature of CDs in the absence of 1D sequence changes. However, two neo‐CDs were identified in the junction region of the rearranged genome fragments by NeoLoopFinder,^[^ ^21^ ^]^ which were located in the junction region of the 48.28M and 75.28M breakpoints, respectively. One of the neo‐CD involved the *MAPK4*, and the other one involved the *DCC* and *CTDP1* (Figure 6g and Figure S9a, Supporting Information). Subsequently, we analyzed the expression of the *MAPK4*, *DCC*, and *CTDP1* genes within the neo‐CD range and found that the transcription of all three genes was extremely low and did not exhibit any significant changes (Table S26, Supporting Information). This may be related to the fact that enhancers marked by H3K27ac within the neo‐CD region had no significant alterations in their activities (Figure 6g and Figure S9a, Supporting Information). The above findings suggest that CD can reduce the disruption of 3D genomic organization caused by complex chromosomal rearrangements, maintaining the basic architecture of the chromatin and stabilizing the expression of genes within the CDs. This may be an intermediate protective mechanism by which chromatin can limit the disruption of gene expression by SVs and could be the result of adaptive selection under natural stresses during evolution.

Indeed, rather than being restricted to chromosomal rearrangement junction regions, extensive ectopic chromosomal rearrangements may also affect long‐range gene regulation throughout chromosome 18 by altering the spatial location of *cis*‐regulatory elements. Therefore, we screened the entire set of differentially expressed genes across chromosome 18. To identify differential gene expression related to chromosome rearrangements, we excluded those genes with no significant difference in expression or low self‐expression level (FPKM < 1) in BxPC3 and PANC1. Finally, a total of 51 candidate rearrangement‐related differentially expressed genes were identified, of which 39 showed higher expression in pancreatic cancer than in adjacent normal tissues and 28 were significantly related to poor prognosis (Figure S9b and Table S27, Supporting Information). Notably, both *MYO5B* and *VPS4B* within the rearrangement region were upregulated in pancreatic cancer and associated with poor prognosis (Figure 6h,i, and Figure S9c,d, Supporting Information). Similarly, *DSC2*, *DSG2*, and *LAMA3* were also found to be highly expressed and significantly associated with poor prognosis in pancreatic cancer (Figure S9c,d, Supporting Information). It has been shown that their encoded proteins are involved in epithelial cell–cell junctions, adhesion, and cell motility and migration and therefore may play an important role in the carcinogenesis and progression of pancreatic cancer.^[^ ^25^, ^49^, ^50^ ^]^

Collectively, our study identified complex chromosomal rearrangements associated with *SMAD4* deletion on chromosome 18 and revealed their effects on 3D genome organization and related gene expression. Moreover, our data further verified the critical role of CDs in maintaining chromatin structural stability. These results provide more clues for understanding the complicated regulatory roles of SVs, 3D chromatin architecture and gene expression changes in the carcinogenesis and progression of pancreatic cancer (Figure 6j).

## 3 Discussion

Recently, with the rapid development and application of TGS and high‐throughput chromatin conformation capture techniques (Hi‐C), increasing evidence has shown that SVs and the 3D genome play critical roles in tumorigenesis and development.^[^ ^13^, ^16^, ^22^, ^23^, ^33^, ^51^ ^]^ However, the spectrum of SVs and overall 3D genome architecture, as well as their dynamic interplay, during the malignant transformation of normal pancreatic ductal epithelium remain largely undefined. In this study, we applied TGS, in situ Hi‐C, and RNA‐seq technologies in combination with data from multiple public databases to perform comprehensive analyses on two PDAC cell lines and a normal immortalized human pancreatic ductal epithelial cell line. This study revealed the signatures of SVs and multidimensional alterations in the spatial organization of chromosomes in PDAC cells and further characterized the complicated interplay of 3D chromosome organization and SVs and their impacts on gene expression. These results could expand our understanding of the complex regulatory network of molecular biology involved in the carcinogenesis and progression of PDAC.

To systematically study SVs in PDAC, we first employed SMRT technology to establish the signatures of SVs in cancer cell lines and identified more than 20 000 SVs. These results fully reflect the substantial advantages of TGS technologies for SV detection and identification.^[^ ^18^ ^]^ Interestingly, up to 23 035 SVs were detected in the immortalized ductal epithelial cell line HPDE6C7, slightly more than the number detected in the cancer cell lines PANC1 and BxPC3. This difference might be related to the process of immortalization of human pancreatic ductal epithelial cells derived from normal adult human pancreatic ducts transfected by the E6E7 gene of human papilloma virus.^[^ ^52^, ^53^ ^]^ In addition, multiple recent studies using TGS have identified more than 20 000 SVs in different normal human genomes,^[^ ^54^, ^55^, ^56^ ^]^ indicating that SVs are polymorphic in the human genome. Such polymorphic SVs can generate novel genomic rearrangements and contribute to the maintenance of genomic diversity.^[^ ^10^ ^]^ Moreover, most of the polymorphic SVs prevalent in the population are not pathogenic.^[^ ^57^ ^]^ To differentiate polymorphic from potentially disease‐causing SVs, it is essential to determine whether an SV occurred de novo or was inherited, as de novo (i.e., disease‐specific) SVs are more frequently associated with the etiology of a disease.^[^ ^57^ ^]^ Therefore, identifying pathogenic SVs will be of great significance and deserve more attention in future studies.

Notably, SVs also have a certain preference in their genomic distribution. First, at the linear genomic level (1D), we found that SVs were mainly distributed in intergenic and intronic regions and less distributed in exonic regions, indicating that most SVs did not alter coding sequences directly, which in turn maintains the evolutionary stability of the human genome. Second, at the 3D genome level, SVs tended to be enriched in compartment A and at the boundaries of CDs, whereas SVs in compartment B and inside CDs were relatively rare, suggesting that SV occurrence and formation may be influenced by 3D genomic organization, while SVs may exert a pathogenic effect by remodeling the genome architecture.^[^ ^10^, ^39^ ^]^ Consequently, in addition to changing the gene dosage in linear genomic exonic regions,^[^ ^58^ ^]^ SV primarily produces pathogenic effects by altering the spatial organization of chromatin to interfere with the positioning and/or copy number of regulatory elements, i.e., exerting position effects.^[^ ^10^ ^]^ Through these position effects, SV may affect the expression of genes that are distant from SV breakpoints and participate in carcinogenesis and progression. For example, deletions and duplications can not only alter the dosage of *cis*‐regulatory elements but also affect their spatial positional distribution, which in turn may affect gene expression through higher‐order chromatin organization of the locus. Similarly, inversions and translocations may affect gene expression and the pathogenic potential of SVs by disrupting the native enhancer regions and CDs or creating novel ones,^[^ ^10^ ^]^ in addition to by disrupting coding sequences or producing fusion transcripts.

Previous studies have shown that SVs on the cross‐CDB remove the isolation effect of the original CCCTC‐binding factor (CTCF)‐related boundary elements, trigger the relocation of enhancers, and may affect enhancer–promoter communication, leading to aberrant gene expression.^[^ ^35^, ^59^, ^60^ ^]^ Deletion and duplication, as the most common simple SVs, have been shown to induce the fusion of adjacent CDs and neo‐CD formation, respectively, in numerous tumors.^[^ ^26^, ^38^, ^51^, ^60^ ^]^ Although similar phenomena were observed in our results, the actual situation was far more complicated than expected. We observed partial cross‐CDB SVs that did not result in corresponding CD fusions or neo‐CD formation. We speculate that this may be related to SV frequency or copy number variations in different cell lines, as Hi‐C data are derived from the average value of a specific cell population. Fused CDs or neo‐CDs within the entire cell population might be masked to varying degrees if the chromosomes of heterogenous cancer cells do not undergo deletion or duplication of CTCF‐associated boundary insulators. Accordingly, our understanding of the true nature of chromatin domains is probably obscured by intercellular genomic heterogeneity in population‐averaged data. The recent development of single‐cell Hi‐C is exciting, as this technology is expected to solve this issue.^[^ ^61^, ^62^ ^]^ More importantly, our study revealed that the structural disturbance in CDs caused by cross‐CDB deletions appeared to be confined to adjacent CDs, indicating that CDs tended to restrict the impact of SVs to the greatest extent and thereby maintained the stability of the overall 3D chromatin architecture. This is consistent with the evolutionarily conserved features of CDs found in the study, which might be the result of adaptive selection under natural evolutionary stress. Therefore, cross‐CDB SVs may affect gene regulation by disrupting the 3D structure of adjacent CDs. However, the subsequent self‐protection mechanism limits the further influence of SVs, indicating that the process of pancreatic carcinogenesis and development involves the dynamic interplay and complex regulation of multiple mechanisms.

Previous studies have identified the loss of *CDKN2A* on chromosome 9 and *SMAD4* on chromosome 18 as important drivers of pancreatic carcinogenesis and progression, with homozygous deletion being a major cause of inactivation of these two key tumor suppressor genes.^[^ ^63^ ^]^ In this study, we first confirmed the presence of *CDKN2A*‐ and *SMAD4*‐related homozygous deletions in PDAC cells using TGS. Then, through Hi‐C sequencing, we found that the homozygous deletion was accompanied by ectopic chromosome rearrangement. Furthermore, we revealed the effects of this complex chromosome rearrangement on 3D genome organization and gene expression alterations, providing new biological insights and potential therapeutic possibilities for understanding the carcinogenesis and progression of PDAC. First, we identified a homozygous *CDKN2A‐CDKN2B‐MTAP* codeletion along with tandem duplications of the flanking regions on chromosome 9 in the PANC1 cell line. This complex SV led directly to the loss of the *CDKN2A* tumor suppressor signaling pathway and significantly upregulated the expression of the *MIR31HG* gene in its neighboring region. This could be the result of cross‐CDB deletion‐related adjacent CD fusion and enhancer hijacking,^[^ ^60^, ^64^, ^65^ ^]^ as well as of an increase in gene dosage at the 1D level caused by tandem duplications within CDs. In practice, deletions and tandem duplications of specific cancer‐related genes are also quite common in other tumors.^[^ ^63^ ^]^ These results indicate that SV can not only alter gene dosage at the 1D level but also regulate the expression of cancer‐related genes through 3D position effects, which collectively contribute to pancreatic carcinogenesis and progression. Previous studies have shown that *MIR31HG* can promote cancer in a variety of solid tumors.^[^ ^44^, ^45^, ^46^ ^]^ Therefore, the development of targeted therapeutic strategies may have good prospects in clinical applications.

Our study also showed that the deletion of a region on chromosome 18 of BxPC3 cells produced an unexpected result on Hi‐C, i.e., the interaction between the regions flanking the lost *SMAD4* gene was not enhanced but basically disappeared, which could not be explained by simple deletion. Further localized scaffold rearrangements on chromosome 18 confirmed the complex ectopic rearrangements of the genome, including the *SMAD4* loss region. Recently, an increasing number of studies have shown that extensive chromosomal rearrangement caused by complex SV is a critical mechanism involved in the genetic instability of PDAC.^[^ ^4^, ^66^, ^67^ ^]^ These findings challenge the current model of PDAC tumorigenesis and provide novel insights into the mutational processes that give rise to these aggressive tumors. However, these studies have focused on the alterations of 1D chromosome structure and their effects on the regulation of gene expression. Based on this knowledge, we further analyzed the chromatin interactions in the rearranged regions of chromosome 18 and found that the interaction frequencies did not change significantly within the rearranged regions that did not involve the CD boundary, suggesting that the presence of an isolated boundary may be crucial for maintaining the stability of CD organization. This is because genomic rearrangements that do not involve boundaries are more likely to alter gene dosage within CDs,^[^ ^10^ ^]^ not the ectopic contacts between adjacent CDs. However, enhanced interactions and neo‐CDs were observed in the junction loci of the chromosomal rearranged segments, but the expression of the *MAPK4*, *DCC*, and *CTDP1* genes within the neo‐CD region did not change dramatically, indicating that neo‐CD formation is not always associated with gene expression changes, especially when *cis‐*regulatory elements remain stable.^[^ ^21^, ^68^ ^]^ Accordingly, gene regulation in PDAC genomes is sophisticated and influenced by multiple factors. Disruption of chromatin folding domains caused by chromosome rearrangement may contribute to gene expression changes, but this is not always the case.^[^ ^69^ ^]^

Next, we analyzed the differentially expressed genes associated with genomic rearrangements across the entirety of chromosome 18 and found that the vast majority of them (39/51) were upregulated in PDAC and were significantly related to poor prognosis. *DSC2* and *DSG2* are typical examples. As an important component of the desmosome and the most widely distributed isoform of desmocollin (DSC), desmocollin 2 (DSC2) has been demonstrated to be essential for the adhesion of epithelial cells and serves as a vital regulator in signaling processes such as epithelial morphogenesis, differentiation, wound healing, cell apoptosis, migration, and proliferation.^[^ ^70^ ^]^ In addition, the desmoglein 2 (DSG2) gene product is a calcium‐binding transmembrane glycoprotein component of desmosomes in vertebrate epithelial cells. A recent study suggested that *DSG2* could promote the carcinogenesis and progression of squamous cell carcinoma by enhancing exosome synthesis and secretion.^[^ ^71^ ^]^ Consequently, these chromosomal rearrangements associated with differential gene expression may serve as prognostic markers and potential therapeutic targets for PDAC in the future. In short, complex genomic rearrangements that occur on the chromosome may affect cancer‐related genes by altering the 3D genome organization and participate in the carcinogenesis and progression of PDAC. Our results provide a new and high‐dimensional perspective for a more comprehensive understanding of the molecular biological processes involved in pancreatic carcinogenesis and development. However, why chromosomal rearrangements occur so frequently in PDAC remains difficult to answer; it is speculated that this may be a result of selective adaptation exerted by extensive desmoplasia.

Notably, an SV manifests as a “junction” between two “breakpoints” in the genome. Only when TGS sequencing reads cross and cover the two breakpoints of the SVs can the SV be identified by TGS SV calling software. In this study, we failed to identify another two large deletions related to chromosome rearrangements not involving *SMAD4* using this software. However, this information could be easily obtained by using a Hi‐C heatmap, suggesting that the current TGS algorithms have limitations in identifying complex SVs associated with chromosome rearrangement. Therefore, the combined application of TGS SV calls, Hi‐C interactive data, and even bionano data and/or genome assembly algorithms are necessary approaches and effective strategies to accurately interpret complex genomic SVs for PDAC in the future.^[^ ^55^ ^]^

In summary, our research applied multiomics techniques to establish the signatures of SVs and 3D genome architecture and characterize the dynamic interplay between them in PDAC. Furthermore, the impact of homozygous deletion of two key driver genes, *CDKN2A* and *SMAD4*, on 3D chromatin folding domains and the expression of related genes in the carcinogenesis and progression of PDAC were specifically elucidated. These findings provide a new spatial perspective toward a comprehensive understanding of the functions and pathogenic mechanisms of SVs in pancreatic carcinogenesis and development. However, it must be acknowledged that there are also some limitations in this study, such as the representativeness of the cell lines and the limitations of TGS technology. Therefore, the conclusions of this study still need to be validated with more basic experiments and in clinical samples. In short, the current research based on high‐dimensional genome provides a genome‐wide resource and might contribute to identifying new molecular markers or potential targets, which is of great practical significance to raise the therapeutic challenges of PDAC with an extremely poor prognosis.

## 4 Experimental Section

### Cell Lines and Culture

Human pancreatic ductal epithelial cells (HPDE6‐C7) and the human pancreatic cancer cell lines PANC1 and BxPC3 were obtained from the American Type Culture Collection (ATCC) (https://www.atcc.org/). All cell lines were cultured under recommended conditions, and PANC1/BxPC3 was authenticated by high‐resolution small tandem repeat profiling. Transcriptome cluster analysis was performed on three cell lines in the CCLE+GSE97003 database, which matched well with the public database.

### Identification of Structural Variations

Genomic DNA was extracted from the cell lines BxPC3, PANC1, and HPDE6C7 using a QIAamp DNA Mini Kit/DNeasy Plant Mini Kit1 (QIAGEN). The integrity of the DNA was determined with an Agilent 4200 Bioanalyzer (Agilent Technologies, Palo Alto, CA). 8 µg of genomic DNA was sheared using g‐Tubes (Covaris) and concentrated with AMPure PB magnetic beads. Each SMRT bell library was constructed using the Pacific Biosciences SMRTbell Template Prep Kit 1.0. The constructed library was size‐selected by the Sage ELF BluePippin system for molecules 8–12 and 14–17 kb, followed by primer annealing and the binding of the SMRT bell templates to polymerases with the DNA Polymerase Binding Kit. Sequencing was carried out for 30 h on the Pacific Bioscience Sequel II platform by Annoroad Genomics. NGMLR (https://github.com/philres/ngmlr) was used to perform the alignment with default parameters. Sniffles^[^ ^18^ ^]^ were used to determine the structural variation by using default parameters and cancer‐specific structural variation was identified by BEDTools.^[^ ^72^ ^]^ The SV classification algorithm was comprehensively defined in another study.^[^ ^73^ ^]^ Cancer genomes were shaped with both simple SVs and complex SVs. In this work, the complex SVs were defined as local assemblies that were made up of multiple SV junctions from different genomic locations on the reference genome, while the simple SV assemblies were defined as assemblies that only contain single junction event.

### Hi‐C Reads Mapping and Normalization

Clean reads were mapped to the *Homo sapiens* genome assembly (hg19) using Bowtie2 (v2.3.4).^[^ ^74^ ^]^ An optimized and flexible pipeline filtered out unmapped, multimapped, or invalid paired‐end reads by Hi‐C Pro.^[^ ^75^ ^]^ Only uniquely valid paired‐end reads were retained for analyses. The interaction matrices at various resolutions (i.e., with the genome partitioned into bins of different sizes) were constructed using HiC‐Pro software (v2.7.1) with default settings.^[^ ^75^ ^]^ Hi‐C interaction matrices were constructed with bin sizes of 1 Mb, 100, 40, 20, 10, and 5 kb at the genome‐wide level following the methods of HiC‐Pro software (v2.7.1) with default settings.^[^ ^75^ ^]^ Briefly, an improved computational efficiency ICE^[^ ^75^ ^]^ (Iterative Correction) (https://github.com/seqyuan/iced) method was utilized to remove potential Hi‐C interaction bias. The genome‐wide Hi‐C resolution values were calculated based on the interaction maps according to previously published definitions.^[^ ^74^ ^]^

### Identification of A/B Compartments

Briefly, the expected score was calculated within each matrix using loess smoothed averaging over the intrachromosomal interactions. Then, the observed/expected ratio of intra‐interaction matrices was obtained. Next, a Pearson's correlation matrix was constructed reflecting the chromosomal interactions for each pair of bins, which was used for principal component analysis. More details can be found in the Supporting Information.

### Identification of CDBs

HiCDB was applied to identify CDBs. The matrix at 10 kb resolution was used as input and HiCDB was run with default parameters. After the boundaries were found, the local relative insulation (LRI) score of each boundary was compared between each sample. When one sample's boundary region LRI score was twice that of the other samples, it was considered to be a specific boundary.^[^ ^36^ ^]^

### Identification of Loops

Genome‐wide chromatin loops were identified using Hi‐C Computational Unbiased Peak Search (HiCCUPS) as part of the Juicer package using 5 kb bins and default parameters.^[^ ^76^ ^]^ To find loops specific to one sample, the observed/expected ratio of the two ends of the loop for each sample was first calculated. Then, a comparison was made. If one sample's observed/expected ratio was twice that of the other sample, then the loop was considered to be specific to that sample.

### RNA‐seq

Total RNA of three cell lines was extracted by the TRIzol method, and libraries were constructed according to a standard protocol (Illumina) and sequenced on the Illumina HiSeq X‐ten system. Three biological replicates were conducted for each library. The resulting filtered reads were aligned against the hg19 reference genome using HISAT2^[^ ^77^ ^]^ with default parameters (v2.1.0), and the expression level of each gene was normalized by the method developed by Traver Hart,^[^ ^78^ ^]^ which was based on the fragments per kilobase per million mapped fragments (FPKM) values. Genes were divided into three groups: highly expressed genes (above the mean), intermediately expressed genes (between the median and the mean), and low expressed genes (below the median). Differentially expressed genes (DEGs) were identified with the DESeq2^[^ ^79^ ^]^ package. Genes with a Benjamini–Hochberg adjusted *q*‐value < 0.05 and an absolute log2‐fold change ≥ 1 were considered differentially expressed.

### ChIP‐seq and Data Analysis

ChIP‐seq reads (NCBI PRJEB27863)^[^ ^73^ ^]^ were aligned to the reference genome using bowtie2^[^ ^74^ ^]^ software, and only unique and nonduplicated mapped reads were used for the downstream analysis. The read coverage and depth were calculated by SAMtools.^[^ ^80^ ^]^ To examine the reproducibility of the ChIP‐seq experiments, deeptools was used to generate the correlation plot for all samples, including input samples. Signal track files in BigWig format were generated using deeptools^[^ ^72^ ^]^ bamCoverage function and were normalized to 1 million reads for visualization. DeepTools was also used to plot the gene body and flanking region heatmap graph using the normalized signal intensity. MACS2 was used to call peaks, followed by peak annotation using bedtools.^[^ ^72^ ^]^ Differential analysis between cancer and normal samples was conducted using bedtools. Functional analysis, such as GO and KEGG for differential peak‐related genes, was performed with in‐house scripts.

Enrichment was assessed using deepTools2 (v3.1.2)^[^ ^81^ ^]^ with default parameters.

### The Distribution of SVs along the 3D Genome

The dynamic chromosome regions were obtained through the comparison of cancer and healthy cell lines. By comparing BXPC3 (or PANC1) to the HPDE6C7 cell line, the chromosome regions could be divided into stable A/B compartments, A‐B compartments, and B‐A compartments. By comparing BXPC3 (or PANC1) to the HPDE6C7 cell line, the chromosome regions could be divided into cancer‐gained TAD boundaries, cancer‐lost TAD boundaries, and stable TAD boundaries. Cancer‐specific SVs referred to those occurring in BXPC3 (or PANC1) but not in HPDE6C7. Cancer‐specific SVs and dynamic chromosome regions were identified by BEDTools. To explore the distribution of SVs, the density of SVs in different chromosome regions, including A/B compartments, TADs, and TAD boundaries was compared. The density of SVs was defined as the number of SVs divided by the length of each chromosome region. SV enrichment was evaluated by comparing the proportion of SVs falling in the region of interest to that in the background, which was performed via the prop.test function in R. The background density referred to the number of SVs divided by the length of the whole genome.

### GO Analysis

A GO method was applied for functional enrichment analysis of the biological processes of the identified genes of interest, such as genes in A/B switch areas, differential loops, or CDs. For each GO term, a *p*‐value was obtained corresponding to a single, independent test and then the BH method was used to correct the *p* values.^[^ ^82^ ^]^

### Neo‐CDs Identification

To identify new CDs in chromosome rearrangement areas, NeoLoopFinder was applied to help find newly emerged CDs in areas that had inversions, translocations, and deletions.^[^ ^21^ ^]^

### Data Visualization

Integrative Genomics Viewer (IGV) was employed to interactively explore large comprehensive genomic data and visualize structural variations as described.^[^ ^83^ ^]^ Annoroad Browser (https://github.com/Spartanzhao/Annoroad‐OMIC‐Viz) was used to produce the track profiles in joint multiomics visualization of the data. Other codes for data processing and visualization are available at https://github.com/Spartanzhao/code_for_Advanced_Science_pancreatic_cell_Line_Hi‐C.

### Statistical Analysis

The rank sum test and Fisher's test were applied to determine the relationship between Hi‐C, transcriptome, and SV data. For gene expressional comparison, data were shown as the interquartile range (IQR) with the median, and *p*‐values were obtained by Wilcoxon rank‐sum test or Kruskal–Wallis test. Constituent ratios were compared by chi‐square test or Fisher's exact test. For biological process enrichment analysis, *p* values were obtained by Fisher's exact test using EnrichR. For Kaplan–Meier survival analysis, the *p* value was obtained by Cox regression in R (version 3.6.3). The values were considered significantly different at *p* < 0.05.

## Conflict of Interest

The authors declare no conflict of interest.

## Author Contributions

Y.D., Z.G., and Z.L. contributed equally to this work. Y.D. and C.W. conceived, designed, and supervised the study with input from H.C. and Z.Y. Y.D., H.C., Z.Y., and Y.Z. designed and performed most of the computational analyses with the help from X.Z., Z.G., and Z.L. Z.G., Z.L., and Y.Z. designed and performed most of the experiments with the help of Z.Y. and Y.D. Y.D., Z.G., and Z.L. wrote the manuscript. All the authors discussed the results and commented on the manuscript. X.B. critically revised the manuscript. H.C. and C.W. were working group or project leader.

## Supporting information

Supporting Information

Supplemental Table 1

Supplemental Table 2

Supplemental Table 3

Supplemental Table 4

Supplemental Table 5

Supplemental Table 6

Supplemental Table 7

Supplemental Table 8

Supplemental Table 9

Supplemental Table 10

Supplemental Table 11

Supplemental Table 12

Supplemental Table 13

Supplemental Table 14

Supplemental Table 15

Supplemental Table 16

Supplemental Table 17

Supplemental Table 18

Supplemental Table 19

Supplemental Table 20

Supplemental Table 21

Supplemental Table 22

Supplemental Table 23

Supplemental Table 24

Supplemental Table 25

Supplemental Table 26

Supplemental Table 27

## Data Availability

All raw and processed sequencing data generated in this study have been submitted to the NCBI Gene Expression Omnibus (https://www.ncbi.nlm.nih.gov/geo/) under accession code GSE185069. Biological material used in this study can be obtained from authors upon request. We used Annoroad Browser (https://github.com/Spartanzhao/Annoroad‐OMIC‐Viz) to produce the track profiles in joint multi‐omics visualization of the data.

## References

[1] R. L. Siegel , K. D. Miller , H. E. Fuchs , A. Jemal , Ca‐Cancer J. Clin. 2021, 71, 7. 33433946 10.3322/caac.21654

[2] J. D. Mizrahi , R. Surana , J. W. Valle , R. T. Shroff , Lancet 2020, 395, 2008. 32593337 10.1016/S0140-6736(20)30974-0

[3] L. Rahib , B. D. Smith , R. Aizenberg , A. B. Rosenzweig , J. M. Fleshman , L M. Matrisian , Cancer Res. 2014, 74, 2913. 24840647 10.1158/0008-5472.CAN-14-0155

[4] F. Notta , M. Chan‐Seng‐Yue , M. Lemire , Y. Li , G. W. Wilson , A. A. Connor , R. E. Denroche , S.‐B. Liang , A. M. K. Brown , J. C. Kim , T. Wang , J. T. Simpson , T. Beck , A. Borgida , N. Buchner , D. Chadwick , S. Hafezi‐Bakhtiari , J. E. Dick , L. Heisler , M. A. Hollingsworth , E. Ibrahimov , G. Ho Jang , J. Johns , L. G. T. Jorgensen , C. Law , O. Ludkovski , I. Lungu , K. Ng , D. Pasternack , G. M. Petersen , et al., Nature 2016, 538, 378. 27732578 10.1038/nature19823 PMC5446075

[5] A. Maitra , R. H. Hruban , Annu. Rev. Pathol.: Mech. Dis. 2008, 3, 157. 10.1146/annurev.pathmechdis.3.121806.154305 PMC2666336 18039136

[6] J. Weischenfeldt , O. Symmons , F. Spitz , J. O. Korbel , Nat. Rev. Genet. 2013, 14, 125. 23329113 10.1038/nrg3373

[7] Lupski , R. James , Environ. Mol. Mutagen. 2015, 56, 419. 25892534 10.1002/em.21943 PMC4609214

[8] G. Macintyre , B. Ylstra , J. D. Brenton , Trends Genet. 2016, 32, 530. 27478068 10.1016/j.tig.2016.07.002

[9] J. Weischenfeldt , O. Symmons , F. Spitz , J. O. Korbel , Nat. Rev. Genet. 2013, 14, 125. 23329113 10.1038/nrg3373

[10] M. Spielmann , D. G. Lupiáñez , S. Mundlos , Nat. Rev. Genet. 2018, 19, 453. 29692413 10.1038/s41576-018-0007-0

[11] P. H. Sudmant , T. Rausch , E. J. Gardner , R. E. Handsaker , A. Abyzov , J. Huddleston , Y. Zhang , K. Ye , G. Jun , M. Hsi‐Yang Fritz , M. K. Konkel , A. Malhotra , A. M. Stütz , X. Shi , F. Paolo Casale , J. Chen , F. Hormozdiari , G. Dayama , K. Chen , M. Malig , M. J. P. Chaisson , K. Walter , S. Meiers , S. Kashin , E. Garrison , A. Auton , H. Y. K. Lam , X. Jasmine Mu , C. Alkan , D. Antaki , et al., Nature 2015, 526, 75.26432246

[12] J. Huddleston , M. J. P. Chaisson , K. M. Steinberg , W. Warren , K. Hoekzema , D. Gordon , T. A. Graves‐Lindsay , K. M. Munson , Z. N. Kronenberg , L. Vives , P. Peluso , M. Boitano , C.‐S. Chin , J. Korlach , R. K. Wilson , E. E. Eichler , Genome Res. 2017, 27, 677. 27895111 10.1101/gr.214007.116 PMC5411763

[13] E L. Van Dijk , Y. Jaszczyszyn , D. Naquin , C. Thermes , Trends Genet. 2018, 34, 666. 29941292 10.1016/j.tig.2018.05.008

[14] M. Nattestad , S. Goodwin , K. Ng , T. Baslan , F. J. Sedlazeck , P. Rescheneder , T. Garvin , H. Fang , J. Gurtowski , E. Hutton , E. Tseng , C.‐S. Chin , T. Beck , Y. Sundaravadanam , M. Kramer , E. Antoniou , J. D. Mcpherson , J. Hicks , W. R Mccombie , M. C. Schatz , Genome Res. 2018, 28, 1126. 29954844 10.1101/gr.231100.117 PMC6071638

[15] S. Aganezov , S. Goodwin , R. M. Sherman , F. J. Sedlazeck , G. Arun , S. Bhatia , I. Lee , M. Kirsche , R. Wappel , M. Kramer , K. Kostroff , D. L. Spector , W. Timp , W. R Mccombie , M. C. Schatz , Genome Res. 2020, 30, 1258. 32887686 10.1101/gr.260497.119 PMC7545150

[16] A. Rhoads , K. F. Au , Genomics, Proteomics Bioinf. 2015, 13, 278. 10.1016/j.gpb.2015.08.002 PMC4678779 26542840

[17] Y. Sakamoto , L. Xu , M. Seki , T. T. Yokoyama , M. Kasahara , Y. Kashima , A. Ohashi , Y. Shimada , N. Motoi , K. Tsuchihara , S. S. Kobayashi , T. Kohno , Y. Shiraishi , A. Suzuki , Y. Suzuki , Genome Res. 2020, 30, 1243. 32887687 10.1101/gr.261941.120 PMC7545141

[18] F. J. Sedlazeck , P. Rescheneder , M. Smolka , H. Fang , M. Nattestad , A. Von Haeseler , M. C. Schatz , Nat. Methods 2018, 15, 461. 29713083 10.1038/s41592-018-0001-7 PMC5990442

[19] N. De Leeuw , T. Dijkhuizen , J. Y. Hehir‐Kwa , N. P. Carter , L. Feuk , H. V. Firth , R. M. Kuhn , D. H. Ledbetter , C. L. Martin , C. M. A. Van Ravenswaaij‐Arts , S. W. Scherer , S. Shams , S. Van Vooren , R. Sijmons , M. Swertz , R. Hastings , Hum. Mutat. 2012, 33, 930. 26285306 10.1002/humu.22049 PMC5027376

[20] J. R. Lupski , C. A. Wise , A. Kuwano , L. Pentao , J. T. Parke , D. G. Glaze , D. H. Ledbetter , F. Greenberg , P. I. Patel , Nat. Genet. 1992, 1, 29. 1301995 10.1038/ng0492-29

[21] X. Wang , J. Xu , B. Zhang , Ye Hou , F. Song , H. Lyu , F. Yue , Nat. Methods 2021, 18, 661. 34092790 10.1038/s41592-021-01164-w PMC8191102

[22] A. R Barutcu , B. R. Lajoie , R. P. Mccord , C. E. Tye , D. Hong , T. L. Messier , G. Browne , A. J. Van Wijnen , J. B. Lian , J. L. Stein , J. Dekker , A. N. Imbalzano , G. S. Stein , Genome Biol. 2015, 16, 214. 26415882 10.1186/s13059-015-0768-0 PMC4587679

[23] P. Wu , T. Li , R. Li , L. Jia , P. Zhu , Y. Liu , Q. Chen , D. Tang , Y. Yu , C. Li , Nat. Commun. 2017, 8, 1937. 29203764 10.1038/s41467-017-01793-w PMC5715138

[24] L. A. E. Nagai , S.‐J. Park , K. Nakai , BMC Med. Genomics 2019, 11, 127. 30894186 10.1186/s12920-018-0437-8 PMC7402584

[25] M. Yang , M. Vesterlund , I. Siavelis , L. H. Moura‐Castro , A. Castor , T. Fioretos , R. Jafari , H. Lilljebjörn , D. T. Odom , L. Olsson , N. Ravi , E. L. Woodward , L. Harewood , J. Lehtiö , K. Paulsson , Nat. Commun. 2019, 10, 1519. 30944321 10.1038/s41467-019-09469-3 PMC6447538

[26] A. Kloetgen , P. Thandapani , P. Ntziachristos , Y. Ghebrechristos , S. Nomikou , C. Lazaris , X. Chen , H. Hu , S. Bakogianni , J. Wang , Yi Fu , F. Boccalatte , H. Zhong , E. Paietta , T. Trimarchi , Y. Zhu , P. Van Vlierberghe , G. G. Inghirami , T. Lionnet , I. Aifantis , A. Tsirigos , Nat. Genet. 2020, 52, 388. 32203470 10.1038/s41588-020-0602-9 PMC7138649

[27] K. C. Akdemir , V. T. Le , S. Chandran , Y. Li , R. G. Verhaak , R. Beroukhim , P. J. Campbell , L. Chin , J. R. Dixon , P. A Futreal , Nat. Genet. 2020, 52, 294.32024999

[28] N. Sidiropoulos , B. R. Mardin , F. Rodríguez‐Gonzalez , S. Garg , J. Weischenfeldt , Genome Res. 2021, 32, 643. 10.1101/gr.275790.121 PMC8997353 35177558

[29] J. R. Dixon , J. Xu , V. Dileep , Y. Zhan , F. Song , V. T. Le , G. G. Yardımcı , A. Chakraborty , D. V. Bann , Y. Wang , R. Clark , L. Zhang , H. Yang , T. Liu , S. Iyyanki , L. An , C. Pool , T. Sasaki , J. C. Rivera‐Mulia , H. Ozadam , B. R. Lajoie , R. Kaul , M. Buckley , K. Lee , M. Diegel , D. Pezic , C. Ernst , S. Hadjur , D T. Odom , J. A. Stamatoyannopoulos , et al., Nat. Genet. 2018, 50, 1388. 30202056 10.1038/s41588-018-0195-8 PMC6301019

[30] J. G. Tate , S. Bamford , H. C. Jubb , Z. Sondka , D. M. Beare , N. Bindal , H. Boutselakis , C. G. Cole , C. Creatore , E. Dawson , P. Fish , B. Harsha , C. Hathaway , S. C. Jupe , C. Y. Kok , K. Noble , L. Ponting , C. C. Ramshaw , C. E. Rye , H. E. Speedy , R. Stefancsik , S. L. Thompson , S. Wang , S. Ward , P. J. Campbell , S. A. Forbes , Nucleic Acids Res. 2019, 47, D941. 30371878 10.1093/nar/gky1015 PMC6323903

[31] Z. Sondka , S. Bamford , C. G. Cole , S. A. Ward , I. Dunham , S. A. Forbes , Nat. Rev. Cancer 2018, 18, 696. 30293088 10.1038/s41568-018-0060-1 PMC6450507

[32] M. A. Nagai , Biomed. Rep. 2016, 4, 275. 26998263 10.3892/br.2016.580 PMC4774362

[33] P. C. Taberlay , J. Achinger‐Kawecka , A. T. L. Lun , F. A. Buske , K. Sabir , C. M. Gould , E. Zotenko , S. A. Bert , K. A. Giles , D. C. Bauer , G. K. Smyth , C. Stirzaker , S. I. O'donoghue , S. J. Clark , Genome Res. 2016, 26, 719. 27053337 10.1101/gr.201517.115 PMC4889976

[34] Y. Gong , C. Lazaris , T. Sakellaropoulos , A. Lozano , P. Kambadur , P. Ntziachristos , I. Aifantis , A. Tsirigos , Nat. Commun. 2018, 9, 542. 29416042 10.1038/s41467-018-03017-1 PMC5803259

[35] M. H. Nichols , V. G. Corces , Cell Rep. 2021, 35, 109330. 34192544 10.1016/j.celrep.2021.109330 PMC8265014

[36] F. Chen , G. Li , M. Q. Zhang , Y. Chen , Nucleic Acids Res. 2018, 46, 11239. 30184171 10.1093/nar/gky789 PMC6265446

[37] W. A. Flavahan , Y. Drier , B. B. Liau , S. M. Gillespie , A. S. Venteicher , A. O. Stemmer‐Rachamimov , M. L. Suvà , B. E. Bernstein , Nature 2016, 529, 110. 26700815 10.1038/nature16490 PMC4831574

[38] M. Franke , D. M. Ibrahim , G. Andrey , W. Schwarzer , V. Heinrich , R. Schöpflin , K. Kraft , R. Kempfer , I. Jerković , W.‐L. Chan , M. Spielmann , B. Timmermann , L. Wittler , I. Kurth , P. Cambiaso , O. Zuffardi , G. Houge , L. Lambie , F. Brancati , A. Pombo , M. Vingron , F. Spitz , S. Mundlos , Nature 2016, 538, 265. 27706140 10.1038/nature19800

[39] N. Sidiropoulos , B. R. Mardin , F. G. Rodríguez‐González , I. D. Bochkov , S. Garg , A. M. Stütz , J. O. Korbel , E. L. Aiden , J. Weischenfeldt , Genome research 2022, 32, 643. 35177558 10.1101/gr.275790.121 PMC8997353

[40] S. Bianco , D. G. Lupiáñez , A. M. Chiariello , C. Annunziatella , K. Kraft , R. Schöpflin , L. Wittler , G. Andrey , M. Vingron , A. Pombo , S. Mundlos , M. Nicodemi , Nat. Genet. 2018, 50, 662. 29662163 10.1038/s41588-018-0098-8

[41] L. Huynh , F. Hormozdiari , Genome Biol. 2019, 20, 60. 30898144 10.1186/s13059-019-1666-7 PMC6427865

[42] A. Hayashi , J. Hong , C. A. Iacobuzio‐Donahue , Nat. Rev. Gastroenterol. Hepatol. 2021, 18, 469. 34089011 10.1038/s41575-021-00463-z

[43] K. J. Mavrakis , E. R Mcdonald , M. R. Schlabach , E. Billy , G. R. Hoffman , A. Deweck , D. A. Ruddy , K. Venkatesan , J. Yu , G. Mcallister , M. Stump , R. Debeaumont , S. Ho , Y. Yue , Y. Liu , Y. Yan‐Neale , G. Yang , F. Lin , H. Yin , H. Gao , D. R. Kipp , S. Zhao , J. T. Mcnamara , E. R. Sprague , B. Zheng , Y. Lin , Y. S. Cho , J. Gu , K. Crawford , D. Ciccone , et al., Science 2016, 351, 1208. 26912361 10.1126/science.aad5944

[44] H. Yang , P. Liu , J. Zhang , X. Peng , Z. Lu , S. Yu , Y. Meng , W.‐M. Tong , J. Chen , Oncogene 2016, 35, 3647. 26549028 10.1038/onc.2015.430 PMC4947634

[45] R. Wang , Z. Ma , L. Feng , Y. Yang , C. Tan , Q. Shi , M. Lian , S. He , H. Ma , J. Fang , Mol. Cancer 2018, 17, 162. 30458787 10.1186/s12943-018-0916-8 PMC6247607

[46] J. Chu , J. Jia , L. Yang , Y. Qu , H. Yin , J. Wan , F. He , Biomed. Pharmacother. 2020, 128, 110313. 32502839 10.1016/j.biopha.2020.110313

[47] W. Huang , B. Navarro‐Serer , Y. J. Jeong , P. Chianchiano , L. Xia , C. Luchini , N. Veronese , C. Dowiak , T. Ng , M. A. Trujillo , B. Huang , M. J. Pflüger , A. M. Macgregor‐Das , G. Lionheart , D. Jones , K. Fujikura , K.‐V. Nguyen‐Ngoc , N. M. Neumann , V. P. Groot , A. Hasanain , A. F. Van Oosten , S. E. Fischer , S. Gallinger , A. D. Singhi , A. H. Zureikat , R. E. Brand , M. M. Gaida , S. Heinrich , R. A. Burkhart , J. He , et al., Cancer Res. 2020, 80, 2804. 32376602 10.1158/0008-5472.CAN-19-1523 PMC7335355

[48] J. N. Burton , A. Adey , R. P. Patwardhan , R. Qiu , J. O. Kitzman , J. Shendure , Nat. Biotechnol. 2013, 31, 1119. 24185095 10.1038/nbt.2727 PMC4117202

[49] Z. Hamidov , A. Altendorf‐Hofmann , Y. Chen , U. Settmacher , I. Petersen , T. Knosel , J. Clin. Pathol. 2011, 64, 990. 21725043 10.1136/jclinpath-2011-200099

[50] K. Hütz , J. Zeiler , L. Sachs , S. Ormanns , V. Spindler , Mol. Carcinog. 2017, 56, 1884. 28277619 10.1002/mc.22644

[51] D. Hnisz , A. S. Weintraub , D. S. Day , A.‐L. Valton , R. O. Bak , C. H. Li , J. Goldmann , B. R. Lajoie , Z. P. Fan , A. A. Sigova , J. Reddy , D. Borges‐Rivera , T. I. Lee , R. Jaenisch , M. H. Porteus , J. Dekker , R. A. Young , Science 2016, 351, 1454. 26940867 10.1126/science.aad9024 PMC4884612

[52] T. Furukawa , W. P. Duguid , L. Rosenberg , J. Viallet , D. A. Galloway , M. S. Tsao , Am. J. Pathol. 1996, 148, 1763. 8669463 PMC1861644

[53] Ni Liu , T. Furukawa , M. Kobari , M. ‐. S. Tsao , Am. J. Pathol. 1998, 153, 263. 9665487 10.1016/S0002-9440(10)65567-8 PMC1852927

[54] M. J. P. Chaisson , A. D. Sanders , X. Zhao , A. Malhotra , D. Porubsky , T. Rausch , E. J. Gardner , O. L. Rodriguez , L. Guo , R. L. Collins , X. Fan , J. Wen , R. E. Handsaker , S. Fairley , Z. N. Kronenberg , X. Kong , F. Hormozdiari , D. Lee , A. M. Wenger , A. R. Hastie , D. Antaki , T. Anantharaman , P. A. Audano , H. Brand , S. Cantsilieris , H. Cao , E. Cerveira , C. Chen , X. Chen , C.‐S. Chin , et al., Nat. Commun. 2019, 10, 1784. 30992455 10.1038/s41467-018-08148-z PMC6467913

[55] P. Ebert , P. A. Audano , Q. Zhu , B. Rodriguez‐Martin , D. Porubsky , M. J. Bonder , A. Sulovari , J. Ebler , W. Zhou , R. Serra Mari , F. Yilmaz , X. Zhao , P. Hsieh , J. Lee , S. Kumar , J. Lin , T. Rausch , Y. Chen , J. Ren , M. Santamarina , W. Höps , H. Ashraf , N. T. Chuang , X. Yang , K. M. Munson , A. P. Lewis , S. Fairley , L. J. Tallon , W. E. Clarke , A. O. Basile , et al., Science 2021, 372. 10.1126/science.abf7117 PMC8026704 33632895

[56] Z. Wu , Z. Jiang , T. Li , C. Xie , L. Zhao , J. Yang , S. Ouyang , Y. Liu , T. Li , Z. Xie , Nat. Commun. 2021, 12, 6501. 34764282 10.1038/s41467-021-26856-x PMC8586011

[57] G. M. Cooper , B. P. Coe , S. Girirajan , J. A. Rosenfeld , T. H. Vu , C. Baker , C. Williams , H. Stalker , R. Hamid , V. Hannig , H. Abdel‐Hamid , P. Bader , E. Mccracken , D. Niyazov , K. Leppig , H. Thiese , M. Hummel , N. Alexander , J. Gorski , J. Kussmann , V. Shashi , K. Johnson , C. Rehder , B. C. Ballif , L. G. Shaffer , E. E. Eichler , Nat. Genet. 2011, 43, 838. 21841781 10.1038/ng.909 PMC3171215

[58] M. Lek , K. J. Karczewski , E. V. Minikel , K. E. Samocha , E. Banks , T. Fennell , A. H. O'donnell‐Luria , J. S. Ware , A. J. Hill , B. B. Cummings , T. Tukiainen , D. P. Birnbaum , J. A. Kosmicki , L. E. Duncan , K. Estrada , F. Zhao , J. Zou , E. Pierce‐Hoffman , J. Berghout , D. N. Cooper , N. Deflaux , M. Depristo , R. Do , J. Flannick , M. Fromer , L. Gauthier , J. Goldstein , N. Gupta , D. Howrigan , A. Kiezun , et al., Nature 2016, 536, 285.27535533

[59] Z. Ordulu , T. Kammin , H. Brand , V. Pillalamarri , C. E. Redin , R. L. Collins , I. Blumenthal , C. Hanscom , S. Pereira , I. Bradley , B. F. Crandall , P. Gerrol , M. A. Hayden , N. Hussain , B. Kanengisser‐Pines , S. Kantarci , B. Levy , M. J. Macera , F. Quintero‐Rivera , E. Spiegel , B. Stevens , J. E. Ulm , D. Warburton , L. E. Wilkins‐Haug , N. Yachelevich , J. F. Gusella , M. E. Talkowski , C. C. Morton , Am. J. Hum. Genet. 2016, 99, 1015. 27745839 10.1016/j.ajhg.2016.08.022 PMC5097935

[60] P. A. Northcott , I. Buchhalter , A. S. Morrissy , V. Hovestadt , J. Weischenfeldt , T. Ehrenberger , S. Gröbner , M. Segura‐Wang , T. Zichner , V. A. Rudneva , H.‐J. Warnatz , N. Sidiropoulos , A. H. Phillips , S. Schumacher , K. Kleinheinz , S. M. Waszak , S. Erkek , D. T. W. Jones , B. C. Worst , M. Kool , M. Zapatka , N. Jäger , L. Chavez , B. Hutter , M. Bieg , N. Paramasivam , M. Heinold , Z. Gu , N. Ishaque , C. Jäger‐Schmidt , et al., Nature 2017, 547, 311. 28726821 10.1038/nature22973 PMC5905700

[61] T. Nagano , S. W. Wingett , P. Fraser , Methods Mol. Biol. 2017, 1654, 79. 28986784 10.1007/978-1-4939-7231-9_6

[62] M. Yu , A. Abnousi , Y. Zhang , G. Li , L. Lee , Z. Chen , R. Fang , T. M. Lagler , Y. Yang , J. Wen , Q. Sun , Y. Li , B. Ren , M. Hu , Nat. Methods 2021, 18, 1056. 34446921 10.1038/s41592-021-01231-2 PMC8440170

[63] Y. Li , N. D. Roberts , J. A. Wala , O. Shapira , S. E. Schumacher , K. Kumar , E. Khurana , S. Waszak , J. O. Korbel , J. E. Haber , M. Imielinski , J. Weischenfeldt , R. Beroukhim , P. J. Campbell , Nature 2020, 578, 112.32025012

[64] P. A. Northcott , C. Lee , T. Zichner , A. M. Stütz , S. Erkek , D. Kawauchi , D. J. H. Shih , V. Hovestadt , M. Zapatka , D. Sturm , D. T. W. Jones , M. Kool , M. Remke , F. M. G. Cavalli , S. Zuyderduyn , G. D. Bader , S. Vandenberg , L. A. Esparza , M. Ryzhova , W. Wang , A. Wittmann , S. Stark , L. Sieber , H. Seker‐Cin , L. Linke , F. Kratochwil , N. Jäger , I. Buchhalter , C. D. Imbusch , G. Zipprich , et al., Nature 2014, 511, 428. 25043047 10.1038/nature13379 PMC4201514

[65] J. Weischenfeldt , T. Dubash , A. P. Drainas , B. R. Mardin , Y. Chen , A. M. Stütz , S. M. Waszak , G. Bosco , A. R. Halvorsen , B. Raeder , T. Efthymiopoulos , S. Erkek , C. Siegl , H. Brenner , O. T. Brustugun , S. M. Dieter , P. A. Northcott , I. Petersen , S. M. Pfister , M. Schneider , S. K. Solberg , E. Thunissen , W. Weichert , T. Zichner , R. Thomas , M. Peifer , A. Helland , C. R. Ball , M. Jechlinger , R. Sotillo , et al., Nat. Genet. 2017, 49, 65. 27869826 10.1038/ng.3722 PMC5791882

[66] I. Cortés‐Ciriano , J. J.‐.K. Lee , R. Xi , D. Jain , Y. L. Jung , L. Yang , D. Gordenin , L. J. Klimczak , C.‐Z. Zhang , D. S. Pellman , K. C. Akdemir , E. G. Alvarez , A. Baez‐Ortega , R. Beroukhim , P. C. Boutros , D. D. L. Bowtell , B. Brors , K. H. Burns , P. J. Campbell , K. Chan , K. Chen , I. Cortés‐Ciriano , A. Dueso‐Barroso , A. J. Dunford , P. A. Edwards , X. Estivill , D. Etemadmoghadam , L. Feuerbach , J. L. Fink , M. Frenkel‐Morgenstern , et al., Nat. Genet. 2020, 52, 331.32025003

[67] O. Shoshani , S. F. Brunner , R. Yaeger , P. Ly , Y. Nechemia‐Arbely , D. H. Kim , R. Fang , G. A. Castillon , M. Yu , J. S. Z. Li , Y. Sun , M. H. Ellisman , B. Ren , P. J. Campbell , D. W. Cleveland , Nature 2021, 591, 137. 33361815 10.1038/s41586-020-03064-z PMC7933129

[68] L. Lu , X. Liu , W.‐K. Huang , P. Giusti‐Rodríguez , J. Cui , S. Zhang , W. Xu , Z. Wen , S. Ma , J. D. Rosen , Z. Xu , C. F. Bartels , R. Kawaguchi , M. Hu , P. C. Scacheri , Z. Rong , Y. Li , P. F. Sullivan , H. Song , G.‐L. Ming , Y. Li , F. Jin , Mol. Cell 2020, 79, 521. 32592681 10.1016/j.molcel.2020.06.007 PMC7415676

[69] Y. Ghavi‐Helm , A. Jankowski , S. Meiers , R. R. Viales , J. O. Korbel , E. E. M. Furlong , Nat. Genet. 2019, 51, 1272. 31308546 10.1038/s41588-019-0462-3 PMC7116017

[70] C. Sun , L. Wang , X.‐X. Yang , Y.‐H. Jiang , X.‐L. Guo , Int. J. Biol. Macromol. 2019, 131, 378. 30851326 10.1016/j.ijbiomac.2019.03.041

[71] J. P. Flemming , B. L. Hill , M. W. Haque , J. Raad , C. S. Bonder , L. A. Harshyne , U. Rodeck , A. Luginbuhl , J. K. Wahl , K. Y. Tsai , P. J. Wermuth , A. M. Overmiller , M. G. Mahoney , J. Extracell. Vesicles 2020, 9, 1790159. 32944178 10.1080/20013078.2020.1790159 PMC7480578

[72] A. R. Quinlan , I. M. Hall , Bioinformatics 2010, 26, 841. 20110278 10.1093/bioinformatics/btq033 PMC2832824

[73] F. H. Hamdan , S. A. Johnsen , Proc. Natl. Acad. Sci. U. S. A. 2018, 115, E12343. 30541891 10.1073/pnas.1812915116 PMC6310858

[74] B. Langmead , S. L. Salzberg , Nat. Methods 2012, 9, 357. 22388286 10.1038/nmeth.1923 PMC3322381

[75] N. Servant , N. Varoquaux , B. R. Lajoie , E. Viara , C.‐J. Chen , J.‐P. Vert , E. Heard , J. Dekker , E. Barillot , Genome Biol. 2015, 16, 259. 26619908 10.1186/s13059-015-0831-x PMC4665391

[76] N. C. Durand , M. S. Shamim , I. Machol , S. S. P. Rao , M. H. Huntley , E. S. Lander , E. L. Aiden , Cell Syst. 2016, 3, 95. 27467249 10.1016/j.cels.2016.07.002 PMC5846465

[77] J. Siren , N. Valimaki , V. Makinen , IEEE/ACM Trans. Comput. Biol. Bioinf. 2014, 11, 375. 10.1109/TCBB.2013.2297101 26355784

[78] T. Hart , H. Komori , S. Lamere , K. Podshivalova , D. R. Salomon , BMC Genomics 2013, 14, 778. 24215113 10.1186/1471-2164-14-778 PMC3870982

[79] M. I. Love , W. Huber , S. Anders , Genome Biol. 2014, 15, 550. 25516281 10.1186/s13059-014-0550-8 PMC4302049

[80] H. Li , B. Handsaker , A. Wysoker , T. Fennell , J. Ruan , N. Homer , G. Marth , G. Abecasis , R. Durbin , Bioinformatics 2009, 25, 2078. 19505943 10.1093/bioinformatics/btp352 PMC2723002

[81] F. Ramírez , D. P. Ryan , B. Grüning , V. Bhardwaj , F. Kilpert , A. S. Richter , S. Heyne , F. Dündar , T. Manke , Nucleic Acids Res. 2016, 44, W160. 27079975 10.1093/nar/gkw257 PMC4987876

[82] M. A. Harris , J. Clark , A. Ireland , J. Lomax , M. Ashburner , R. Foulger , K. Eilbeck , S. Lewis , B. Marshall , C. Mungall , J. Richter , G. M. Rubin , J. A. Blake , C. Bult , M. Dolan , H. Drabkin , J. T. Eppig , D. P. Hill , L. Ni , M. Ringwald , R. Balakrishnan , J. M. Cherry , K. R. Christie , M. C. Costanzo , S. S. Dwight , S. Engel , D. G. Fisk , J. E. Hirschman , E. L. Hong , R. S. Nash , Nucleic Acids Res. 2004, 32, D258. 14681407 10.1093/nar/gkh036 PMC308770

[83] J. T. Robinson , H. Thorvaldsdóttir , W. Winckler , M. Guttman , E. S. Lander , G. Getz , J. P. Mesirov , Nat. Biotechnol. 2011, 29, 24. 21221095 10.1038/nbt.1754 PMC3346182

