# Supplementary material for: Dynamic Interplay between Structural Variations and 3D Genome Organization in Pancreatic Cancer
Source: Adv Sci (Weinh). 2022 May 15;9(18):2200818. doi: 10.1002/advs.202200818 (PMC9218654; doi:10.1002/advs.202200818)
Supplement: Supplementary file 1 — Supporting Information [file ADVS-9-2200818-s004.pdf]

ADVANCED SCIENCE
Open Access

Supporting Information

for *Adv. Sci.*, DOI 10.1002/advs.202200818

Dynamic Interplay between Structural Variations and 3D Genome Organization in Pancreatic Cancer

Yongxing Du, Zongting Gu, Zongze Li, Zan Yuan, Yue Zhao, Xiaohao Zheng, Xiaochen Bo, Hebing Chen and Chengfeng Wang**

Supporting Information

Title: Dynamic interplay between structural variations and 3D genome organization in pancreatic cancer

Running title: Carcinogenesis of structural variations in pancreatic cancer

Author: Yongxing Du[1†], Zongting Gu[1†], Zongze Li[1†], Zan Yuan[2], Yue Zhao[2], Xiaohao Zheng[1], Xiaochen Bo[3], Hebing Chen[3*], Chengfeng Wang[1*]

Author Affiliations:

1 Department of Pancreatic and Gastric Surgery, National Cancer Center/Cancer Hospital, Chinese Academy of Medical Sciences and Peking Union Medical College, Beijing 100021, People's Republic of China.

2 Annoroad Gene Technology Co., Ltd, Beijing 100176, People's Republic of China.

3 Department of Biotechnology, Institute of Health Service and Transfusion Medicine, Beijing 100850, People's Republic of China

[†] These authors contributed equally to this work.

[*] **Corresponding author:**

Chengfeng Wang, E-mail: wangchengfeng62@163.com.

Hebing Chen, E-mail: chb-1012@163.com.

Materials and Methods

Hi-C library preparation and sequencing

We used 40 ml 2% formaldehyde solution to crosslink the seed tissue for 15 min at room temperature in vacuum. Then, we added 4.324 ml of 2.5 M Gly to quench the cross-linking reaction. The supernatant and tissues were removed from the precipitate. We kept the tissue in liquid nitrogen. Tissues were resuspended in 25 ml extraction buffer (10 mM Tris-HCl at pH 8, 10 mM $MgCl_2$, 0.4 M sucrose, 0.1 mM phenylmethylsulfonyl fluoride [PMSF], 5 mM b-mercaptoethanol, and 13 protease inhibitors; Roche). Next, the suspension was filtered through Miracloth (Calbiochem). We then performed centrifugation at 4000 rpm at 4°C for 20 min. After that, the pellet was resuspended in 1 ml extraction II buffer (10 mM Tris-HCl pH 8, 1% Triton X-100, 0.1 mM PMSF, 0.25 M sucrose, 10 mM $MgCl_2$, 5 mM mercaptoethanol, and 13 protease inhibitors). Later, the solution was centrifuged for 10 min at 14000 rpm at 4°C. The pellet was resuspended in 300 μl extraction buffer III (10 mM Tris-HCl pH 8, 1.7 M sucrose, 2 mM $MgCl_2$, 1 μL protease inhibitor, 0.1 mM PMSF, 5 mM b-mercaptoethanol, 0.15% Triton X-100). Afterward, another 300 μl of clean

extraction buffer III was loaded and centrifuged at 14000 rpm for 10 min. The supernatant was removed, and the pellet was washed with 500 µl ice-cold 1x CutSmart buffer and centrifuged at 2500 g for 5 min each time. The remaining pellets contained the nuclei. Then, we used restriction enzyme buffer to wash the pellet twice and moved it to a safe-lock tube. The next step was to solubilize the chromatin with dilute SDS and incubate it at 65°C for 10 min. Then, the SDS was quenched by Triton X-100 overnight. Next, the nuclei were digested by 4 cutter restriction enzymes (400 units MboI) at 37°C on a rocking platform. The next step was to mark the DNA ends by biotin-14-dCTP and then perform blunt-end ligation between cross-linked fragments. Thus, the ligation enzyme could ligate the proximal chromatin DNA. We incubated the nuclear complexes with proteinase K at 65°C, and then the nuclear complexes were reverse cross-linked. DNA was then purified by phenol-chloroform extraction. We used T4 DNA polymerase to remove biotin-C from unligated fragments. Next, we used sonication to shear the fragments to 200-600 base pairs and repair the fragments with a mixture of T4 DNA polymerase, Klenow DNA polymerase and T4 polynucleotide kinase. Biotin-labeled DNA fragments were then

enriched by streptavidin C1 magnetic beads. The Hi-C library from the beads was sequenced on the Illumina HiSeq X Ten platform with 150 bp paired-end reads. Raw reads were trimmed to 50 bp and then filtered by fqtools plus (https://github.com/annoroad/fqtools_plus) to discard the reads with adapters (> 5 bp adapter nucleotide) and a high N ratio (>5%) and low-quality reads.

Differential interaction and calculation of chromatin interaction

To analyze the difference between interaction matrices in the BxPC3, PANC1, and HPDE6C7 samples, the different matrices were computed by subtracting the z-score matrices of sample-paired intra-interaction matrices. To measure the distance-dependent decay in the cis-interactions of a genome, we used 1 Mb interaction matrices. The intra-chromosome interaction frequency between each bin with the same distances on the reference genome was calculated as real distance in space (https://github.com/dekkerlab/cworld-dekker). Interaction frequencies were log10 transformed to fit a linear model. The slope of each model was outputted as the corresponding finalized interaction decay exponents (IDE) value (https://github.com/dekkerlab/cworld-dekker).

Identification of A/B compartments

We first calculated the eigenvalues of the first and second principal components for each chromosome. Then, we found the A/B compartments according to the positive and negative eigenvalues of each bin and adjusted by gene density. After that, we checked whether the gene expression level in each type of compartment in each chromosome corresponded to the definition of A/B compartments, in which the A compartment gene expression level was higher and the B compartment gene expression level was lower. We also checked the iced matrices and Pearson's correlation matrices of each chromosome. Finally, we determined which chromosome uses eigen2 values and which chromosome uses eigen1 values in each sample and each chromosome. In addition, we also determined whether -1 was multiplied in each chromosome's eigenvalue. In the end, for each chromosome, the A/B compartments were determined to have higher gene expression level in A compartment and lower gene expression level in B compartment, and we were able to identify different types of compartment interaction patterns in the iced matrix and Pearson's correlation matrix (https://github.com/dekkerlab/cworld-dekker).

Public database analysis

Data of gene expression, mutation, and survival information were obtained from the cBioportal TCGA Pan Cancer Studies or Cancer Cell Line Encyclopedia (Broad, 2019) panel. RNA-seq data were downloaded from the Google Cloud Pilot RNA-sequencing for CCLE and TCGA open-access repository (https://osf.io/gqrz9/). For GSE97003, RNA-seq data were downloaded from GEO (https://www.ncbi.nlm.nih.gov/geo/query/acc.cgi?acc=GSE97003). Raw counts of cell lines were processed log2-transformed normalization followed by Z-score transformation before clustering.

URLs: NCBI, https://www.ncbi.nlm.nih.gov.GEPIA2,http://gepia2.cancer-pku.cn/#general.cBioPortal,https://www.cbioportal.org.GTEX,https://www.gtexportal.org/home/index.html. TCGA, https://www.tcga.org. XENA, https://xena.ucsc.edu. Arrayexpress,https://www.ebi.ac.uk/arrayexpress.CCLE,https://portals.broadinstitute.org/ccle/about.GSE97003,https://www.ncbi.nlm.nih.gov/geo.ExPASy,https://web.expasy.org/cellosaurus. IGV, https://www.igv.org. ATCC, https://genomes.atcc.org/.

Results

Cis and trans interactions in PDAC

We first analyzed the genome-wide interaction ratio of each chromosome in the three cell lines and found that most of the interactions were intrachromosomal, whereas only a few interactions were inter-chromosomal (Supplementary Figure 3b and 3c). Next, we analyzed the correlation between chromosome size, spatial position and inter-chromosomal interactions. We found that small, gene-rich chromosomes (chromosomes 16, 17, 19, 20, 21, 22) preferentially interacted with each other, suggesting that chromosome proximity strongly influenced contact probability (Supplementary Figure 3d), which is consistent with previous studies[1-3]. In addition, heatmap showed some strong abnormal inter-chromosome interactions (Supplementary Fig. 3c), and Circos[4] analysis revealed the top 1000 inter-chromosome interactions at 1M resolution in both cancer cell lines (BxPC3 and PANC1) and the normal HPDE6C7 cell line (Supplementary Fig. 3e). Inter-chromosome translocations normally lead to abnormal strong inter-chromosome

(trans) interactions, the CNVs region often have strong abnormal interactions with others. The top trans-interactions bin pairs of the cancer cells may correspond with these SVs.

Compartment switching

Genome-wide comparative analysis identified common and specific compartment shifts in each PDAC cell line compared with HPDE6C7. The proportions of stable A and B in the genomes of BxPC3 and PANC1 were 33.03% and 29.39%, respectively, and the common A-to-B and B-to-A ratios were 7.8% and 3.53%, respectively (Supplementary Figure 4a). Interestingly, the specific A-to-B and B-to-A switching in BxPC3 cells accounted for 7.45% and 6% of all genome, respectively. Correspondingly, specific A-to-B and B-to-A transitions occurred in 8.46% and 4.33% of the genome in PANC1, respectively, suggesting the cell type specificity of A/B switching.

1. Lieberman-Aiden, E. *et al.* Comprehensive mapping of long-range interactions reveals folding principles of the human genome. *Science* **326**, 289-93 (2009).
2. Krzywinski, M. *et al.* Circos: an information aesthetic for comparative genomics. *Genome Res* **19**, 1639-45 (2009).
3. Nagai, M.A. Pleckstrin homology-like domain, family A, member 1 (PHLDA1) and cancer. *Biomed Rep* **4**, 275-281 (2016).

4. Taberlay, P.C. *et al.* Three-dimensional disorganization of the cancer genome occurs coincident with long-range genetic and epigenetic alterations. *Genome Res* **26**, 719-31 (2016).

Supplementary Figures and figure legends

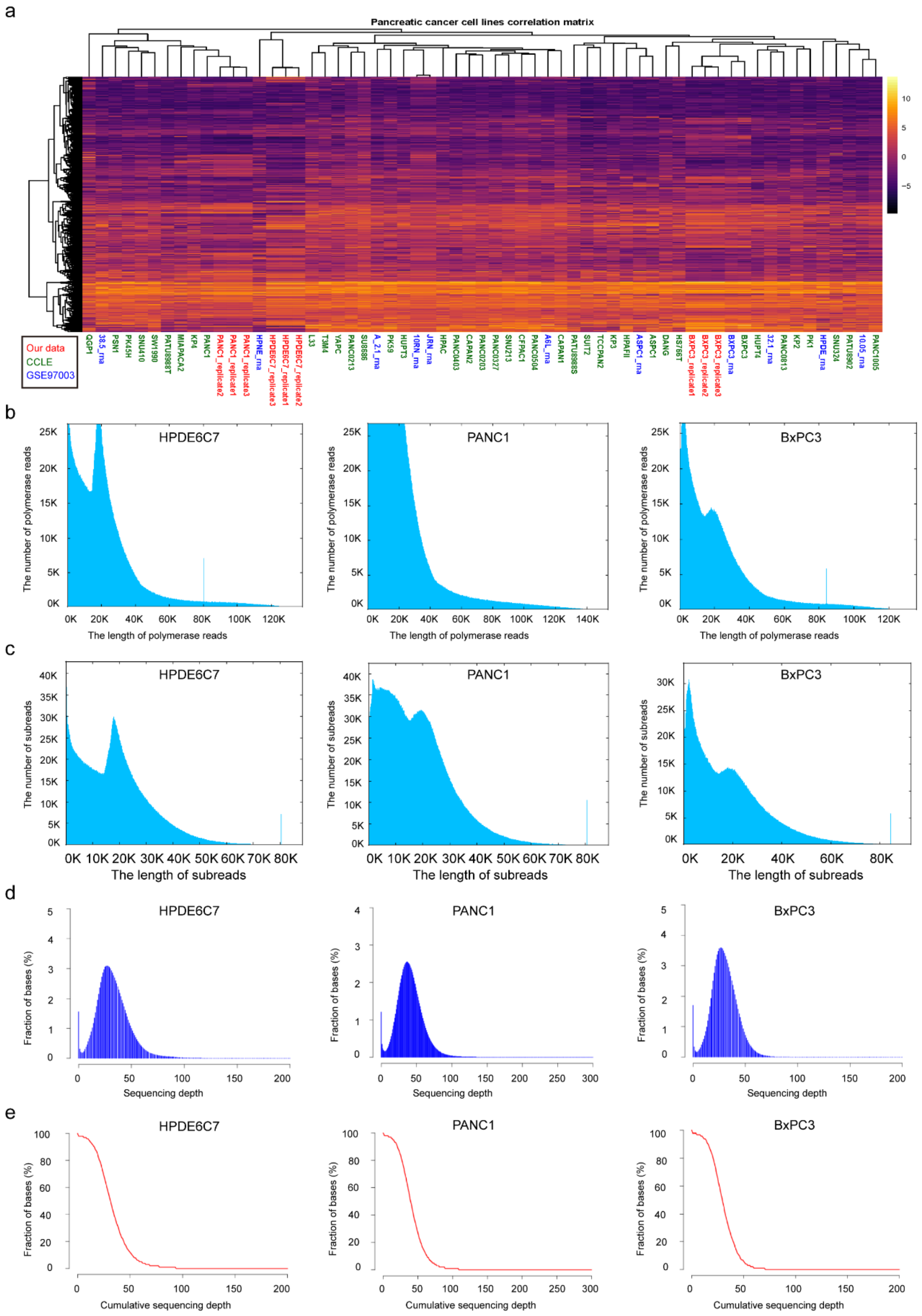

Supplementary Figure 1 | Cell line identification and quality control of long-read sequencing data. a, The gene expression heatmap plot of the 5000 most variable

genes ranked by interquartile range (IQR) across PDAC cell lines for the unsupervised clustering of our data (red), CCLE (green) and GSE97003 (blue). **b**, Histogram showing polymerase read lengths of HPDE6C7, PANC1 and BxPC3. **c**, Histogram showing subread lengths of HPDE6C7, PANC1 and BxPC3. **d**, Fraction of bases as a function of sequencing depth. **e**, Fraction of bases as a function of cumulative sequencing depth.

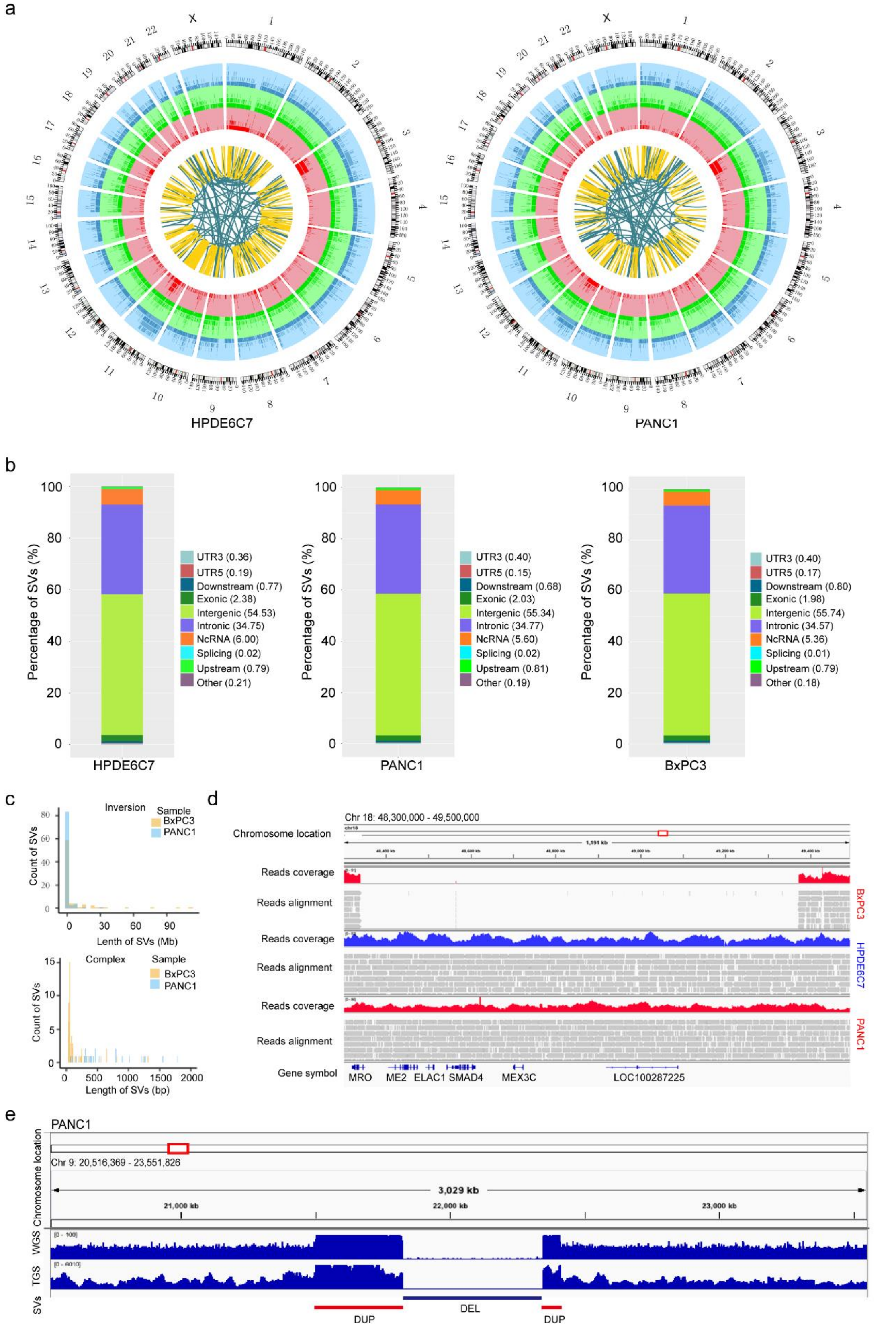

Supplementary Figure 2 | The overall landscape of SVs in PANC1, BxPC3 and

HPDE6C7. a, Circos plots showing the high-confidence SVs detected by Sniffles in HPDE6C7 and PANC1 with 23 chromosomes inputted. The tracks from the outer to the inner circles are the chromosome coordinates, deletions, insertions, duplications and translocations. **b**, Distribution of SVs in different regions of the genome in HPDE6C7, PANC1 and BxPC3. **c**, Specific SV size histogram of PANC1 and BxPC3 found by SMRT variant calling for inversions and complex SVs. **d**, IGV image showing a homozygous deletion of SMAD4 in chromosome 18 of BxPC3. **e**, IGV image showing duplications in the region adjacent to the homozygous deletion covering CDKN2A, CDKN2B, and MTAP in PANC1.

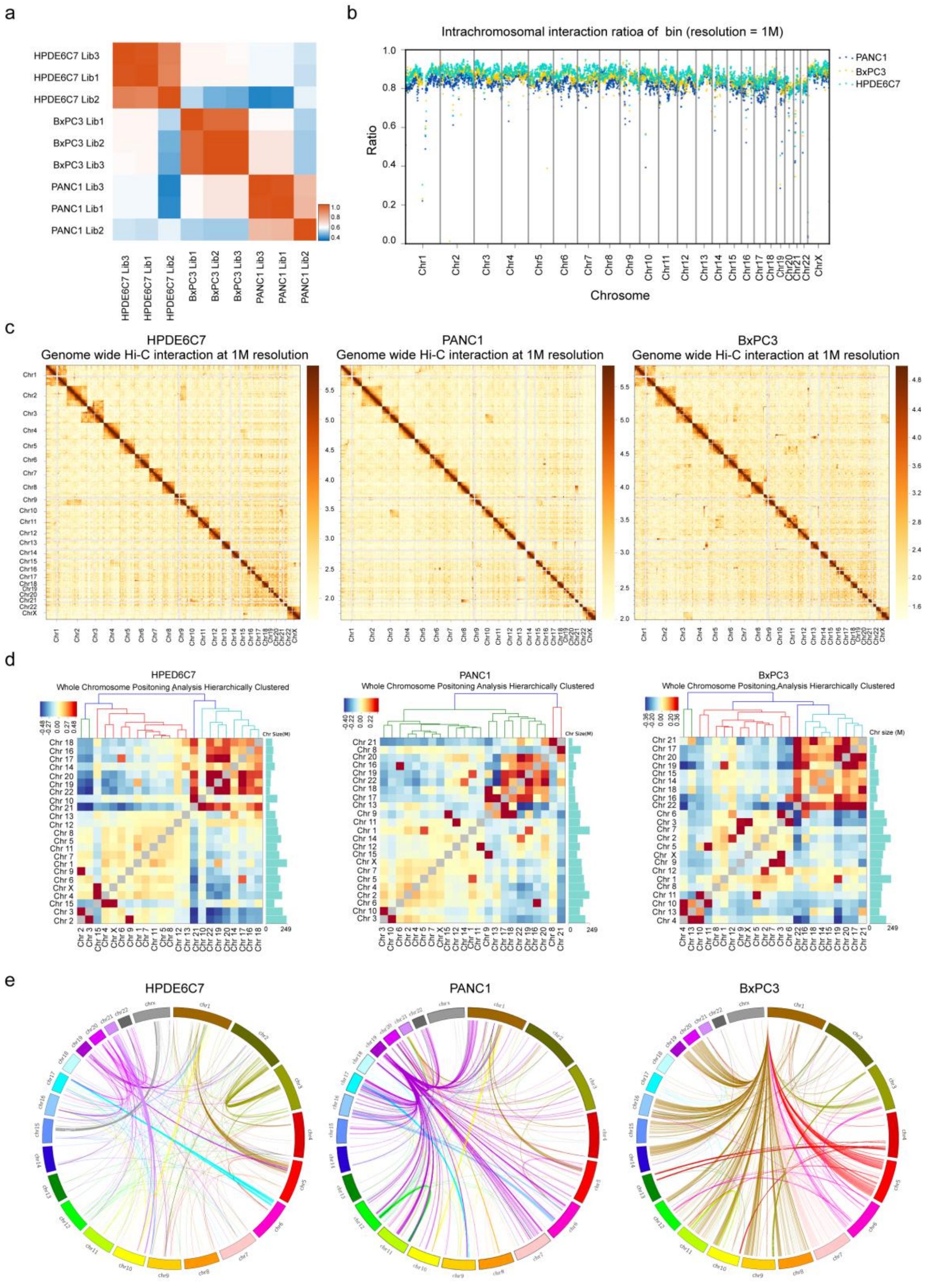

Supplementary Figure 3 | Analysis of whole chromosomal interactions in three different cell lines. a, Hi-C data normalization and initial quality control. Red

indicates stronger interactions, and blue indicates weaker interactions. **b**, Intrachromosomal interaction ratio at 1-Mb resolution in the three cell lines. **c**, Genome-wide all-by-all Hi-C interaction heatmap. **d**, Inter-chromosomal interactions between all pairs of chromosomes in HPDE6C7, PANC1 and BxPC3. Each block represents observed/expected interactions between chromosomes. Red and blue indicate enriched and depleted, respectively. The histogram on the right shows the chromosome size; the coordinates are in M units. **e**, Circos plot showing the top 1000 inter-chromosome interactions at 1M resolution. The curve indicates the position of the 1000 bin pairs with the strongest interactions.

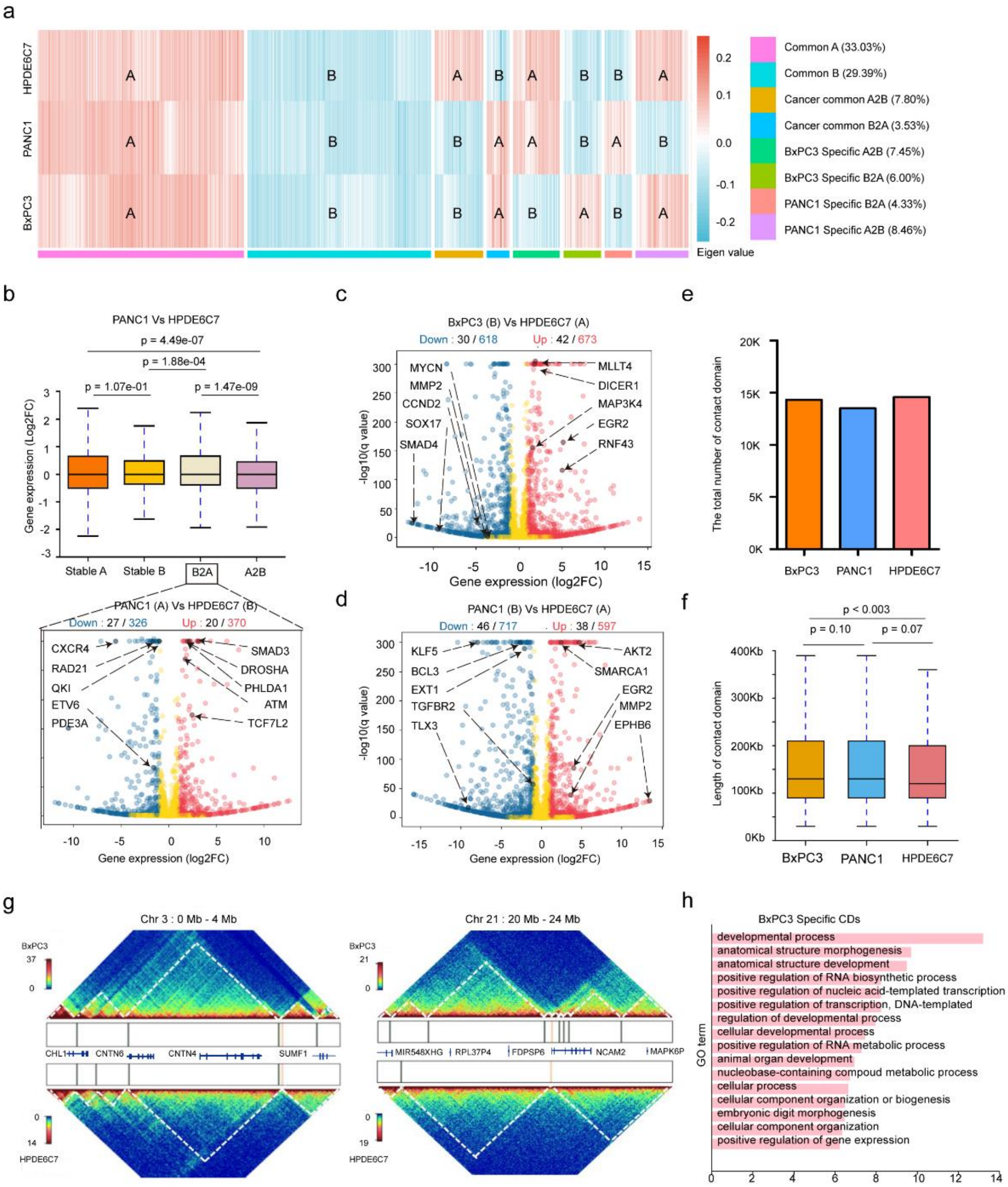

Supplementary Figure 4 | 3D chromatin architecture is remodeled and correlates with gene expression changes in human PDAC. a, Compartment analysis using eigenvalues on all Hi-C datasets. **b**, Top: Box plots showing the gene expressional comparison in different compartments between PANC1 and HPDE6C7. The box represents the interquartile range (IQR), with the centerline denoting the median, and

the whiskers extend to 1.5 times the IQR (or to the maximum/minimum if < 1.5 × IQR). Bottom: Volcano plots show the number of differentially expressed genes (blue) and cancer-related genes (black) among them in the B-to-A compartment shift region. The genes indicated by the black arrow are examples of upregulated (red on the right) or downregulated (blue on the left) cancer-related genes (|Log2FC|>1 and adjusted p value<0.05). Gene expression was compared as Log2FC (BxPC3/HPDE6C7) with P-values obtained by the Wilcoxon rank-sum test. **c**, **d**, Volcano plots show the number of differentially expressed genes (blue) and cancer-related genes (black) among them in the A-to-B compartment shift region comparing BxPC3, PANC1 and HPDE6C7. The genes indicated by the black arrow are examples of upregulated (red, right) or downregulated (blue, left) cancer-related genes (|Log2FC|>1 and adjusted p value<0.05). Gene expression was compared as Log2FC (BxPC3/HPDE6C7) with the P-value obtained by the Wilcoxon rank-sum test. **e**, Histograms showing the number of CDs from HiCDB interaction matrices at 10-kb resolution in the three cell lines. **f**, Box plots represent the length of CDs from HiCDB interaction matrices at 10-kb resolution in the three cell lines. The box represents the interquartile range (IQR),

with the centerline denoting the median, and the whiskers extend to 1.5 times the IQR (or to the maximum/minimum if < 1.5 × IQR). P-values were obtained by Wilcoxon rank-sum test. **g**, Examples of CD alterations in regions (left chr 3: 0-4 Mb, right chr 21: 20-24 Mb) comparing BxPC3 and HPDE6C7. The vertical bars in the box between the heatmaps represent CDBs. The genes involved in the region are marked. **h**, Biological process enrichment of differentially expressed genes located in specific CDs of BxPC3. The P values were obtained via Fisher's exact test using Enrich R. GO, Gene Ontology**.**

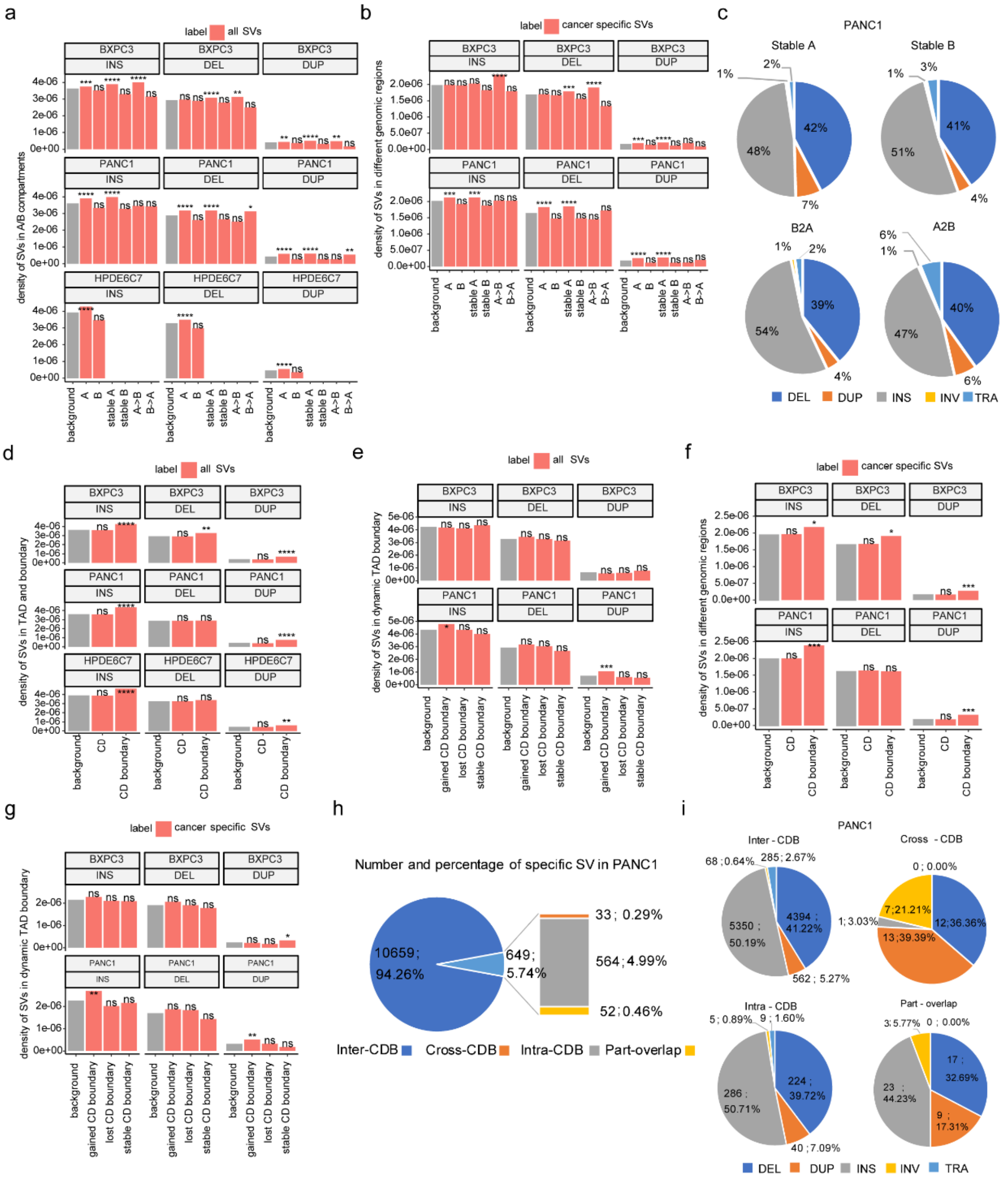

Supplementary Figure 5 | Distributions of structural variations among 3D genome architectures. a, b, The density of SVs (insertions, deletions and duplications) in A/B compartments. Enrichment was evaluated by comparing the proportion of SVs falling in the region of interest and the proportion of the length of

the region of interest in the whole genome via R’s prop. test. ****$p \leq 0.0001$, ***$p \leq 0.001$, **$p \leq 0.01$, *$p \leq 0.05$. **c**, The proportion of different types of cancer-specific SVs (less than 2 Mb length) in different A/B compartment regions of PANC1. **d**, **e**, The density of SVs (insertions, deletions and duplications) in CDs and CDBs. Enrichment was evaluated by comparing the proportion of SVs falling in the region of interest and the proportion of the length of the region of interest in the whole genome via R’s prop. test. ****$p \leq 0.0001$, ***$p \leq 0.001$, **$p \leq 0.01$, *$p \leq 0.05$. **f**, **g**, The density of SVs (insertions, deletions and duplications) in dynamic CD boundaries. Enrichment was evaluated by comparing the proportion of SVs falling in the region of interest and the proportion of the length of the region of interest in the whole genome via R’s prop. test. ****$p <= 0.0001$, ***$p \leq 0.001$, **$p \leq 0.01$, *$p \leq 0.05$. **h**, Number and percentage of specific SVs in PANC1. **i**, Number and percentage of different types of four categories of cancer-specific SVs in PANC1according to Figure 3d.

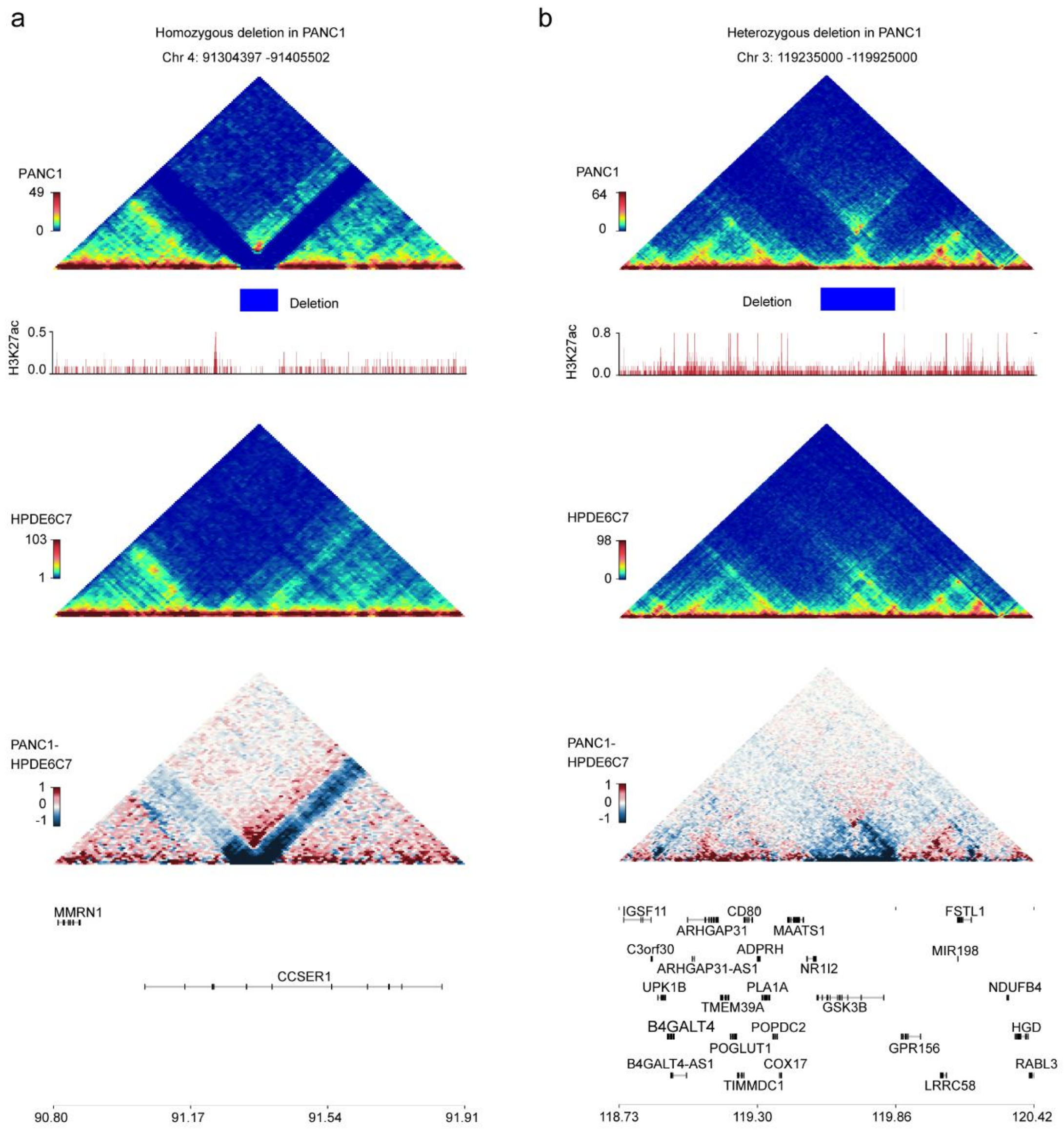

Supplementary Figure 6 | Examples of the impact of cross-CDB deletion frequency on the chromatin folding domain in PANC1. Triangle heatmaps represent chromatin contact frequency, with the top showing PANC1, middle showing HPDE6C7, and bottom showing the subtractive results. Histogram representing roadmap epigenome enhancer activity, marked by H3K27ac, in PANC1 (red). a, Homozygous cross-CDB

deletion is associated with CD fusion. b, No significant enhancement of adjacent CD interactions is observed in the heterozygous cross-CDB deletion.

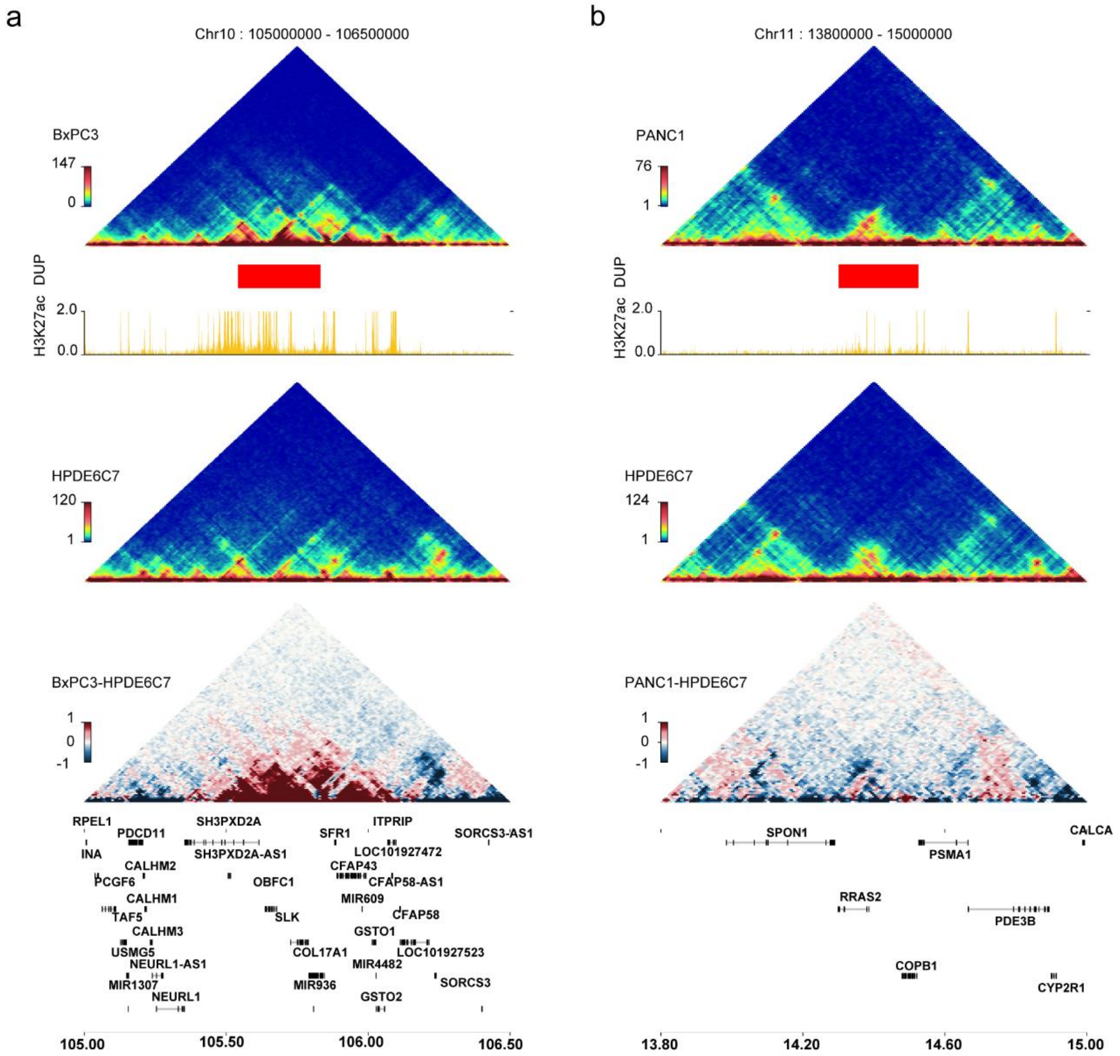

Supplementary Figure 7 | Examples of the impact of cross-CDB duplication frequency on the chromatin folding domain in BxPC3 and PANC1. Triangle heatmaps represent chromatin contact frequency, with the top showing cancer cells, middle showing HPDE6C7, and bottom showing the subtractive results. The

histogram represents roadmap epigenome enhancer activity, marked by H3K27ac, in cancer cells (red). a, Homozygous cross-CDB duplication is associated with neo-CD formation in the BxPC3 cell line. b, No significant enhancement of adjacent CD interactions was observed in the heterozygous cross-CDB duplication in the PANC1 cell line.

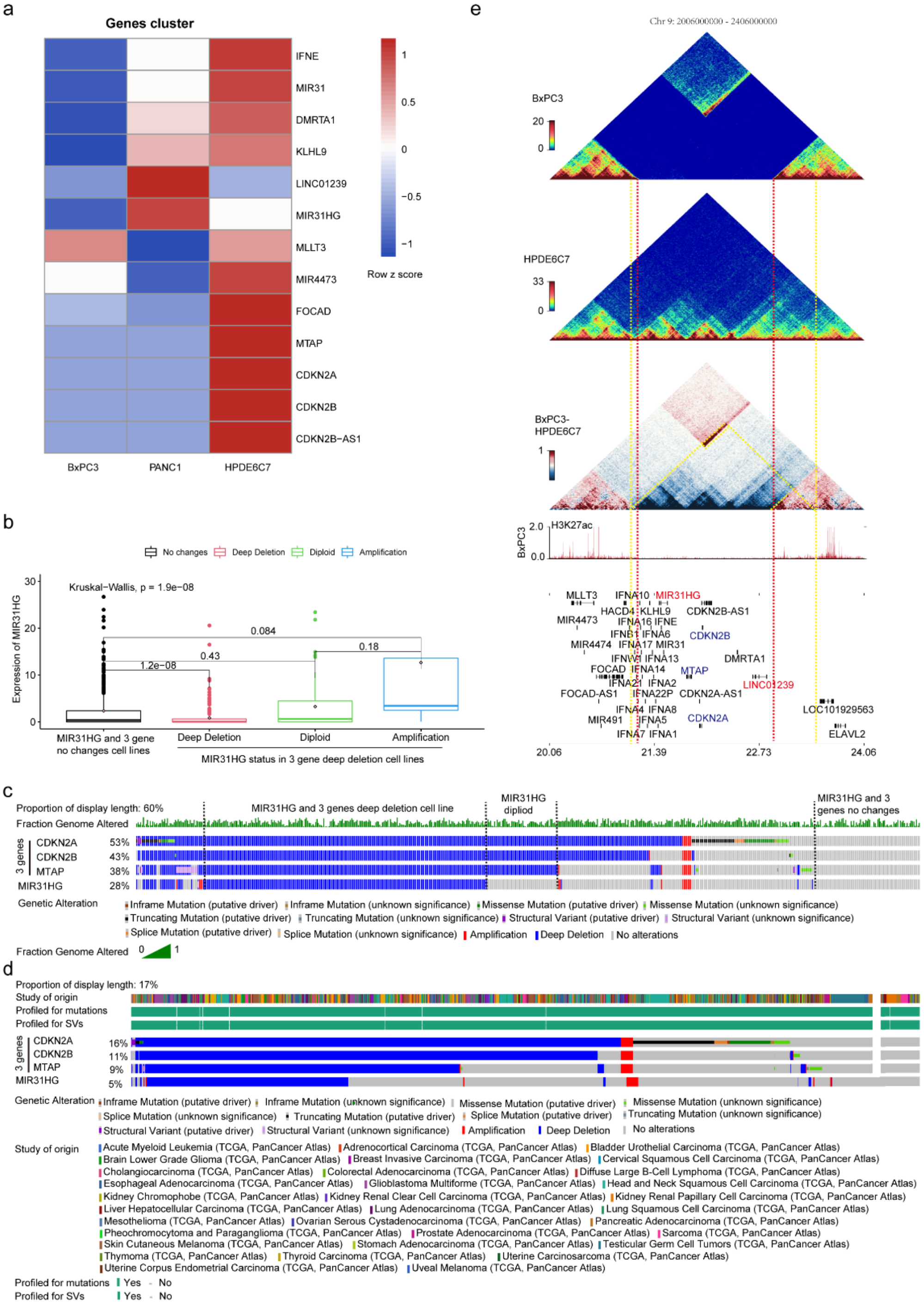

Supplementary Figure 8 | CDKN2A homozygous deletion is associated with MIR31HG upregulation in part through concomitant adjacent genome amplification and CD fusion. a, Heatmap representation of RNA-seq results for

genes in the CDKN2A-CDKN2B-MTAP deletion region corresponding to Figure 5a. The heatmap shows the row z score of FPKM normalized read counts. **b**, MIR31HG expression levels in cases of different mutation states of MIR31HG and the three-gene deletion (CDKN2A, CDKN2B and MTAP) in pancancer cell lines from CCLE. The box represents the interquartile range (IQR), the centerline denotes the median and the whiskers extend to 1.5 times the IQR (or to the maximum/minimum if < 1.5 × IQR). P-value is obtained by Kruskal–Wallis test. **c**, Genetic alteration display of CDKN2A, CDKN2B, MTAP and MIR31HG in 807 cancer cell samples from the CCLE dataset by CBioPortal (see URLs). Limited by page size, only 60% of the primary diagram is presented; this includes all cancer cell lines with deep deletion of MIR31HG and the three genes. **d**, Genetic alterations in CDKN2A, CDKN2B, MTAP and MIR31HG in 8359 pancancer samples from TCGA dataset by CBioPortal (see URLs). Limited by page size, only 17% of the primary diagram is presented; this includes all cancer cell lines with deep deletion of MIR31HG and the three genes. **e**, Diagram showing the impacts of CDKN2A homozygous deletion on 3D chromatin folding domains in BxPC3. Triangle heatmaps represent chromatin contact frequency, with the top

showing BxPC3, middle showing HPDE6C7, and bottom showing the subtractive results. The histogram below represents roadmap epigenome enhancer activity, marked by H3K27ac, in BxPC3. The red dashed line denotes the break points of the homozygous deletion, and the yellow dashed line marks the boundaries of the fused CD. The yellow dashed line in the bottom triangle heatmap indicates the enhanced interactions of the adjacent CD.

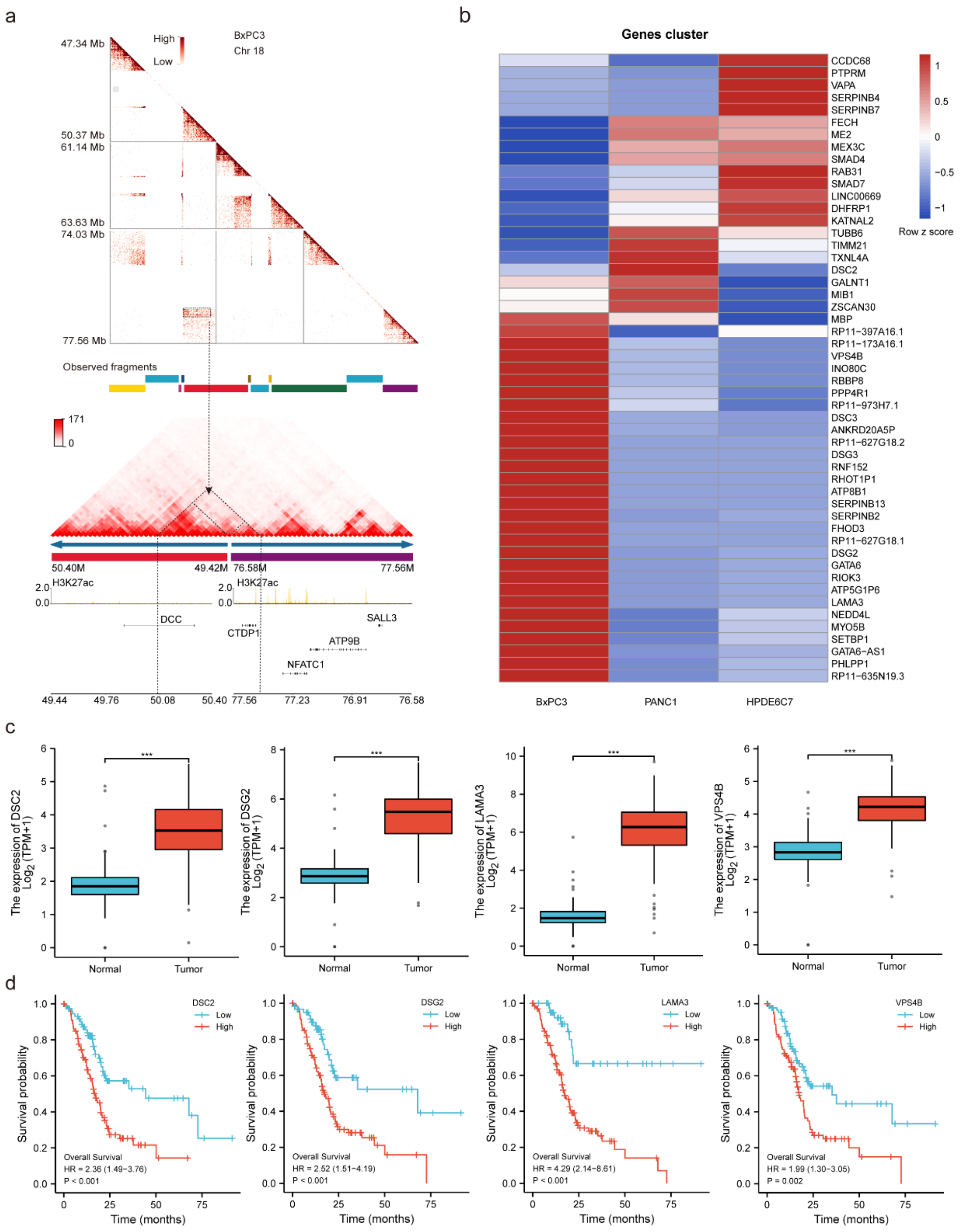

Supplementary Figure 9 | Identification of complex genomic rearrangement associated with SMAD4 deletion in PDAC. a, Enlarged heatmap indicated by triangles and rectangles in Figure 6c. The dashed box shows an example of the aberrant enhanced interaction (neo-CD) in the junction region of rearranged purple

and red genome fragments in chromosome 18 of BxPC3 by NeoLoopFinder. The dashed triangle denotes the neo-CD corresponding to ectopic interactions in the enlarged heatmap with the dashed black arrow indicated. The histograms below represent roadmap epigenome enhancer activity, marked by H3K27ac, in BxPC3 (yellow). **b**, Heatmap representation of RNA-seq results for differentially expressed gene-associated complex rearrangements in chromosome 18 in BxPC3. The heatmap shows the row z score of FPKM normalized read counts. **c**, Expression of DSC2, DSG2, LAMA3, and VPS4B in pancreatic cancer and normal control tissues from TCGA and GTEx (n = 350). The box represents the interquartile range (IQR), the centerline denotes the median and the whiskers extend to 1.5 times the IQR (or to the maximum/minimum if < 1.5 × IQR). P-values were obtained by Wilcoxon rank-sum test. ***p ≤ 0.001. **d**, Kaplan–Meier curves for overall survival according to DSC2, DSG2, LAMA3, and VPS4B expression in the TCGA pancreatic cancer dataset (n = 178). P values were obtained by Cox regression via the R package (version 3.6.3).

Supplementary Tables are attached to supplementary table files.

Supplementary Table 1 | Quality control of long-read sequencing data in HPDE6C7, PANC1 and BxPC3.

Supplementary Table 2 | The number of different types of SVs detected by long-read sequencing in HPDE6C7, PANC1 and BxPC3.

Supplementary Table 3 | The number of different types of cancer-specific SVs detected by long-read sequencing in PANC1 and BxPC3 cells.

Supplementary Table 4 | List of genes directly affected by SVs in exon regions of the genome in HPDE6C7, PANC1 and BxPC3.

Supplementary Table 5 | List of cancer-specific genes directly affected by SVs in exon regions of the genome in PANC1 and BxPC3.

Supplementary Table 6 | List of cancer-specific human cancer genes directly affected by SVs in exon regions of the genome in PANC1 and BxPC3.

Supplementary Table 7 | Comparison of HPDE6C7, PANC1 and BxPC3 by Cellosaurus.

Supplementary Table 8 | Quality control of Hi-C sequencing data.

Supplementary Table 9 | A/B transition in BxPC3 and PANC1 compared with HPDE6C7.

Supplementary Table 10 | Significantly differentially expressed genes in the A/B transition (Log2FC > 1 and q < 0.05).

Supplementary Table 11 | The number and length of CDs called by HiCDB in HPDE6C7, PANC1 and BxPC3 cells.

Supplementary Table 12 | The number and percentage of CDBs called by HiCDB in HPDE6C7, PANC1 and BxPC3 cells.Supplementary Table 13 | Gene list of specific CDs of PANC1 and BxPC3.

Supplementary table 14 | Gene list in shared cancer specific CDBs in BxPC3 and PANC1

Supplementary Table 15 | Specific loops in PANC1 and BxPC3.

Supplementary table 16 | Abnormally Activated Gene List Associated with Neo Enhancer-Promoter Loops in Cancer cell lines.

Supplementary Table 17 | The distribution of cancer-specific SVs in the A/B compartments of PANC1.Supplementary Table 18 | The distribution of cancer-specific

SVs in the A/B compartments of BxPC3.

Supplementary Table 19 | The distribution of cancer-specific SVs in CDs of BxPC3 and PANC1.

Supplementary Table 20 | Cross-CDB-Del and CD fusion in BxPC3 and PANC1.

Supplementary Table 21 | Gene list and expression changes in fused CDs of BxPC3.

Supplementary Table 22 | Gene list and expression changes in fused CDs of PANC1.

Supplementary Table 23 | Gene expression change in the Chr 9 CDKN2A-CDKN2B-MTAP deletion region.

Supplementary Table 24 | MIR31HG expression in the CCLE database.

Supplementary Table 25 | MIR31HG expression in TCGA database.

Supplementary Table 26 | The expression level of three genes in the neo-CD of the rearranged fragment junction region.

Supplementary Table 27 | List of differentially expressed genes in chromosome 18.
